# Species richness and identity both determine the biomass of global reef fish communities

**DOI:** 10.1038/s41467-021-27212-9

**Published:** 2021-11-25

**Authors:** Jonathan S. Lefcheck, Graham J. Edgar, Rick D. Stuart-Smith, Amanda E. Bates, Conor Waldock, Simon J. Brandl, Stuart Kininmonth, Scott D. Ling, J. Emmett Duffy, Douglas B. Rasher, Aneil F. Agrawal

**Affiliations:** 1 Tennenbaum Marine Observatories Network and MarineGEO program, Smithsonian Environmental Research Center, Edgewater, MD 21037 USA; 2 Institute for Marine and Antarctic Studies, University of Tasmania, Hobart, TAS 7001 Australia; 3 Department of Ocean Sciences, Memorial University of Newfoundland, St. John’s, NF A1C 5S7 Canada; 4 Department of Biology, University of Victoria, Victoria, BC V8P 5C Canada; 5 Landscape Ecology, Institute of Terrestrial Ecosystems, ETH Zürich, CH-8092 Zürich, Switzerland; 6 Aquatic Ecology and Evolution, Institute of Ecology and Evolution, University of Bern, Bern, Switzerland; 7 Department of Marine Science, The University of Texas at Austin, Marine Science Institute, Port Aransas, TX 78373 USA; 8 School of Marine Studies, The University of South Pacific, Laucala Bay Road, Suva, Fiji Islands; 9 Bigelow Laboratory for Ocean Sciences, East Boothbay, ME 04544 USA; 10 Department of Ecology & Evolutionary Biology, University of Toronto, Toronto, ON M5S 3B2 Canada

**Keywords:** Community ecology, Ocean sciences, Biodiversity

## Abstract

Changing biodiversity alters ecosystem functioning in nature, but the degree to which this relationship depends on the taxonomic identities rather than the number of species remains untested at broad scales. Here, we partition the effects of declining species richness and changing community composition on fish community biomass across >3000 coral and rocky reef sites globally. We find that high biodiversity is 5.7x more important in maximizing biomass than the remaining influence of other ecological and environmental factors. Differences in fish community biomass across space are equally driven by both reductions in the total number of species and the disproportionate loss of larger-than-average species, which is exacerbated at sites impacted by humans. Our results confirm that sustaining biomass and associated ecosystem functions requires protecting diversity, most importantly of multiple large-bodied species in areas subject to strong human influences.

## Abstract (web summary)

Species identity and richness both contribute biodiversity-ecosystem functioning relationships. Here the authors apply a decomposition approach inspired by the Price equation to a global dataset of reef fish community biomass, finding that increased richness and community compositions favouring large-bodied species enhance biomass.

## Introduction

Extinctions in nature are often biased towards species that are rare and large-bodied^1,2^, yet experiments designed to predict the ecosystem consequences of such extinctions have typically removed species at random^3^. The few experiments that have employed more realistic extinction scenarios by removing rare and functionally-unique species have revealed greater changes in ecosystem functioning than from random losses^4–6^. Observational datasets provide a unique opportunity to examine how the nonrandom loss of species, particularly those that are too large or too rare to feasibly manipulate in experiments, affects ecosystem processes under natural circumstances. Such datasets reflect the cumulative processes—including natural environmental forcing, local losses and gains of species, and anthropogenic impacts—that generate gradients of biodiversity observed in the real world^7^.

Even as observational evidence supports strong links between species richness and ecosystem functioning, specifically biomass production^7^, it remains unclear how much of this relationship stems from reducing the number of species (at random) versus the specific traits proxied by the identities of species. This distinction is important if extinctions are biased towards individual species that contribute disproportionately to functioning, such as large-bodied fishes^8^. Selective removal of such species through overexploitation and sensitivity to human disturbance might be expected to have a larger-than-anticipated influence on community productivity compared to random losses^9^.

Separating the effects of changing species richness and community composition on ecosystem properties in observational data has proved historically challenging^10^. One useful approach is to mathematically partition the total change in an aggregate property, such as community biomass, into component factors^11–15^. Inspired by these earlier approaches, we present a partitioning of the difference in standing stock biomass between pairs of communities—a reference site vs. a comparison site—into five additive components representing the effects of both gains and losses in species richness and composition, and the effects of any remaining influences such as environmental and ecological factors that are not already captured in the elements of community diversity.

**Fig. 1:**
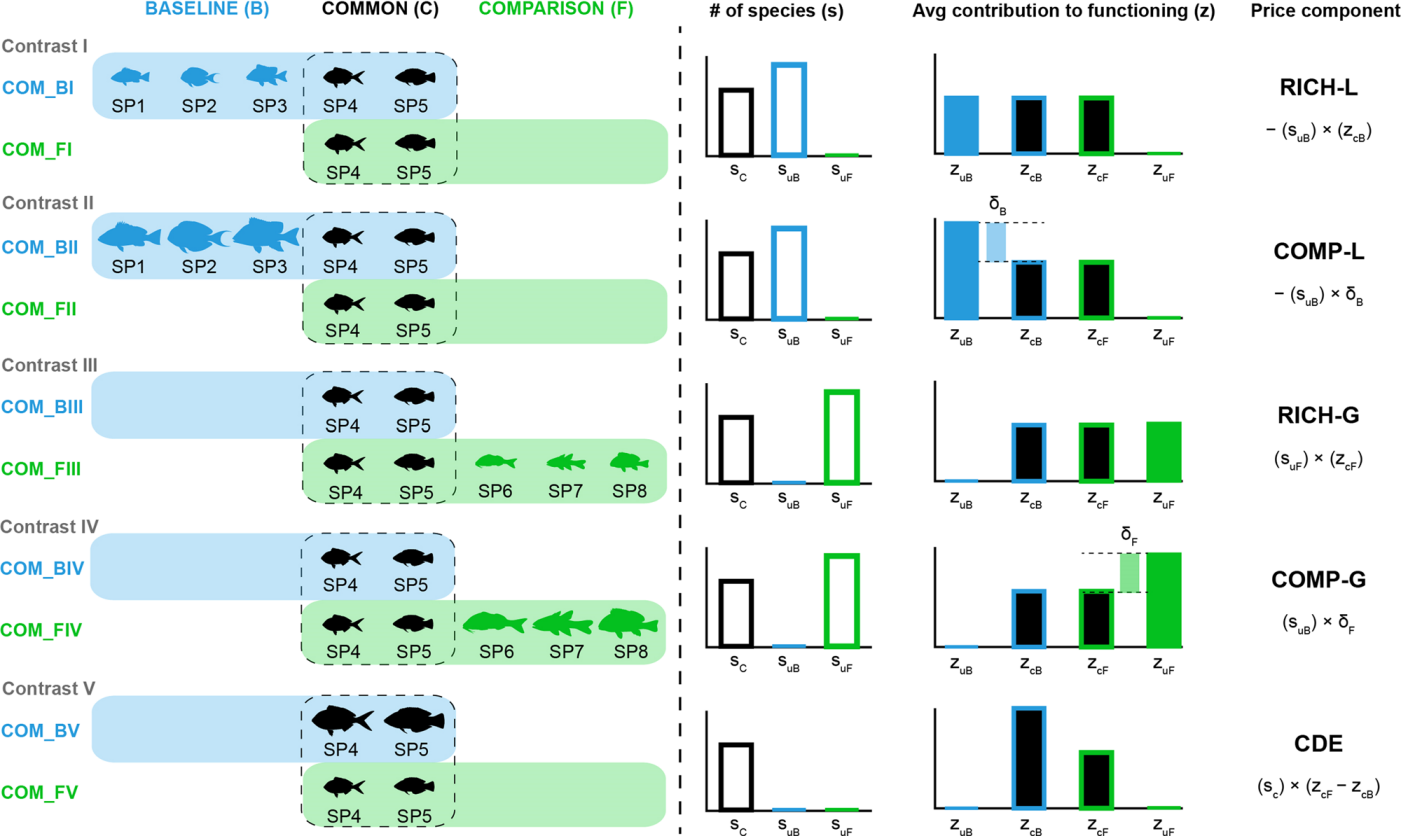
A conceptual representation of our decomposition quantifying the difference in biomass between two sites: a reference or reference site (*B*) and a comparison site (*F*). Notation: S_*c*_, S_*uB*_, and S_uF_ refers to the number of species: in common, unique to *B* and unique to *F*, respectively; and Z_*uB*_, Z_*cB*_, Z_*cF*_, and Z_*uF*_ to average ecological function (in this example, biomass) of species: unique to *B*, common to both sites when present at *B*, common to both sites when present at *F*, and unique to *F*, respectively. The first term, *RICH-L*, reflects loss of species from the reference site that are most like the average of the species that are retained. In Contrast I, the comparison site lacks some species present at the reference site (left column), so the species richness is lower at the comparison site (second column). In this case, the species in common between the two sites have the same average contribution to biomass (per species) as species unique to the reference (i.e., *z*_*cB*_ = *z*_*uB*_, third column). Thus, the difference in biomass between the two sites in Contrast I is entirely captured by the *RICH-L* effect (last column). The second term, *COMP-L*, reflects the loss of biomass beyond what is expected given the number of species lost and average per species contribution of the reference species, i.e., the shared species. Unlike in Contrast I, in the case shown for Contrast II the species unique to the reference site are larger and therefore greater contributors (per species) to total biomass than the species that are shared between sites (${\bar{{{{{{\boldsymbol{z}}}}}}}}_{{{{{{\boldsymbol{uB}}}}}}}{{{{{\boldsymbol{-}}}}}}{\bar{{{{{{\boldsymbol{z}}}}}}}}_{{{{{{\boldsymbol{cB}}}}}}}$ = *δ*_*B*_ > 0). The difference in total biomass between communities in Contrast II would be captured by the *COMP-L* term (last column) in addition to the *RICH-L* term (as shown for Contrast I). The *RICH-G* and *COMP*-*G* terms are analogous to *RICH-L* and *COMP*-*L* but arise from species that are present in the comparison site and absent from the reference (as illustrated in Contrasts III and IV). The final term, the “context-dependent effect” or *CDE*, considers only the species shared among both the reference and comparison sites. Differences in the contributions of shared species among the two sites reflect processes other than changes in richness and composition, such as changes in per species biomass, community size structure, resources, or the abiotic environment (e.g., temperature). In Contrasts I-IV, the shared species did not differ in their average per species contribution between the two sites (i.e., *z*_*uF*_ = *z*_*cB*_) so there was no *CDE*. In Contrast V, the shared species differ in their average per species contribution to biomass, resulting in a nonzero *CDE*. In a real comparison, all five components can occur simultaneously. The five components sum to the observed difference in biomass between the reference and comparison communities.

We must first describe the background of our decomposition (Fig. 1). Species that are absent or “lost” in the comparison community relative to the reference community contribute to the “richness loss” and the “compositional loss” components of the biomass difference, hereafter *RICH-L* and *COMP-L*. To quantify these components, it is necessary to choose a frame of reference for the “expected” biomass loss per absent species, under the null hypothesis that there is nothing unusual about the species that are absent. Here, we use the average biomass of species present in both communities (“shared species”) as this reference value (for more details, see Methods and Supplementary Materials). *RICH-L* is the amount of biomass lost based on the number of species absent from the comparison community weighted by this “expected” biomass per species. The actual loss in biomass at a community level can differ due to deviations in the biomass of the species that are absent compared to the species that are shared, which is captured by the *COMP-L* term. This term reflects the change in biomass between communities that is attributable to the kinds of species that are absent rather than simply the number of absent species.

The third and fourth terms reflect changes in biomass from species present at the comparison site but absent from the reference site (“gained” species): *RICH-G* is the expected gain in biomass given the number of gained species (again based on the average biomass of species shared among both sites), and *COMP-G* reflects any additional biomass change from the gained species being different in average biomass from shared species. The addition of the previous four terms (*RICH-L* + *COMP-L* + *RICH-G* + *COMP-G*) summarizes the total difference in community biomass between the two sites due to both losses and gains in the number (richness) and identities (composition) of species, which we call the “total diversity effect” or *DIV*.

The fifth and final term aims to estimate all additional factors contributing to the difference in biomass between sites that is not due to diversity, broadly speaking. These include, for example, differences in the number of individuals and their sizes, predator–prey interactions, resource availability, habitat quality and complexity, and/or underlying environmental drivers, collectively called the “context-dependent effect” (or *CDE*). The *CDE* term considers only differences in biomass among species shared by both communities rather than species, which are lost or gained between communities. We note that the *CDE* term cannot mechanistically distinguish among these various influences, as has been done using other experimental^16^ and observational datasets^7^, but its relative magnitude can be assessed against other terms to quantify the importance of losses or gains of whole populations (i.e., *RICH* and *COMP*) versus changes in individual biomass or abundance^13^. By extension, environmental factors can also drive changes in species richness and composition, so *CDE* should not be interpreted as exclusively indicative of environmental forcing. Rather, it can be thought of as the cumulative influence of all extrinsic factors on fish community properties that is independent of their realized effects on the observed differences in richness and composition.

**Fig. 2:**
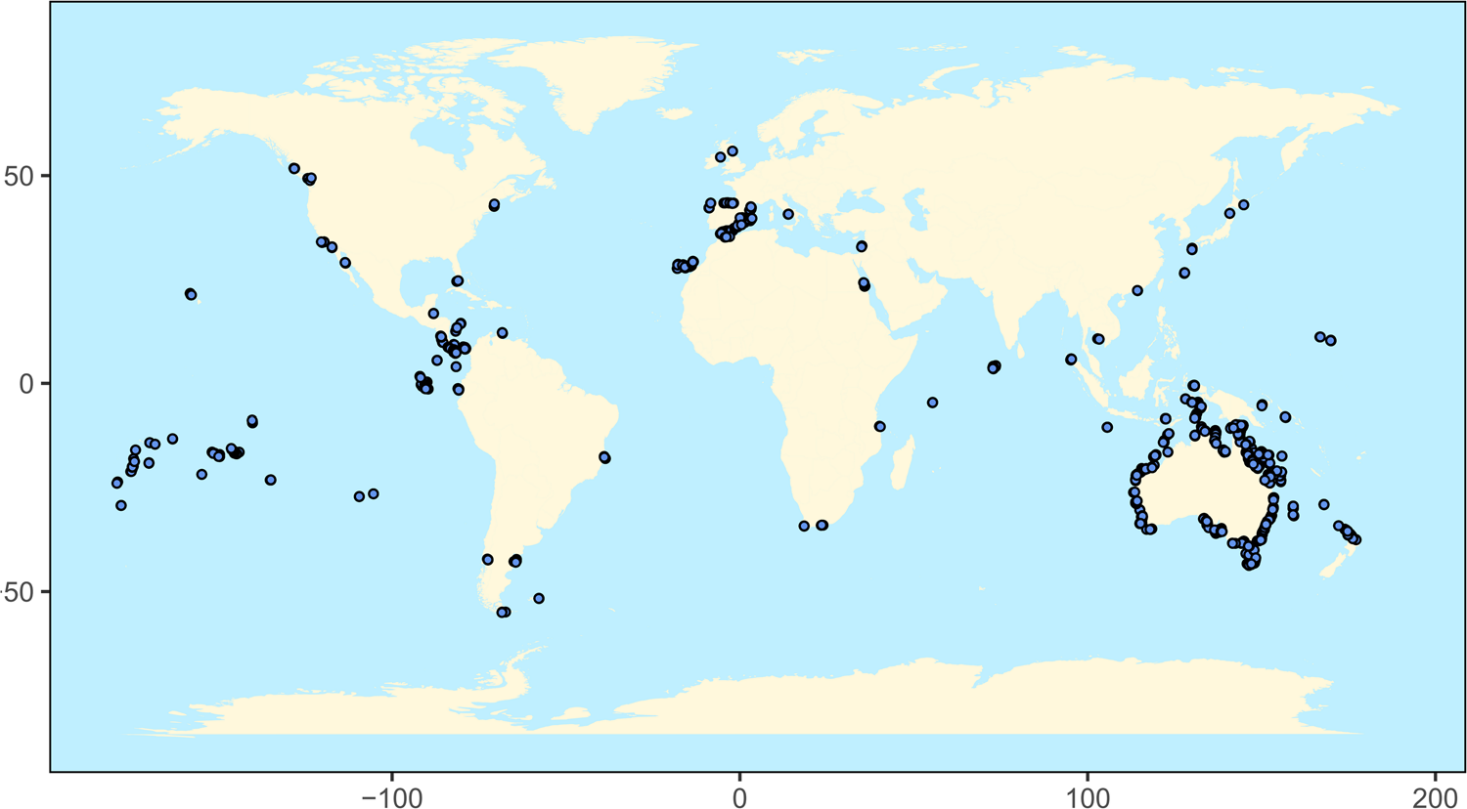
A map of study sites included in the analysis.

Here, we applied this decomposition to quantitatively assess the relative contributions of species richness, composition, and context-dependent effects to reef fish community biomass, a common proxy for secondary production, derived from a fisheries-independent global dataset of underwater visual censuses collected by the Reef Life Survey program (www.reeflifesurvey.com). We selected reefs with the highest biomass to serve as the reference sites, as high fish biomass is a desirable state and often the goal of conservation and restoration efforts. We then compared these high-biomass sites to all other sites within a 100-km radius (representing a potential upper limit on direct dispersal^17^) to determine whether variation in elements of biodiversity contributes to other nearby sites not reaching their biomass potential. We conducted 173 comparisons of reference sites (i.e., those with the highest biomasses) against a total of 2867 comparison sites, with each reference site having on average 16.6 (range = 5–136) comparison sites (Fig. 2). Sites were split near equally between tropical realms (mostly coral-dominated; *n* = 1603) and temperate realms (mostly rocky reefs; *n* = 1437). We go on to show that loss of biomass at the comparison site is most associated with biodiversity loss, which can be further attributed to both losses in the total number of species and the absence of uniquely large contributors to biomass. These effects were most pronounced in areas with high human populations, implicating humans as a primary driver of changes in fish biomass across the seascape.

## Results and discussion

**Fig. 3:**
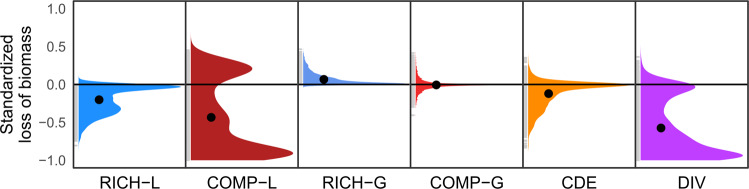
Declines in fish biomass between sites are driven primarily by loss of species (*RICH-L*) and compositional losses (*COMP-L*). Panels show the frequency distributions of each component based on differences between sites with the highest biomass and other nearby sites. Values have been standardized to the interval (−1, 1). Black points represent the mean of all observations ±95% confidence intervals (which are too small to be observed). RICH richness, COMP composition, L loss, G gain, CDE context-dependent effect, DIV total diversity effect (= *RICH-L* + *COMP-L* + *RICH-G* + *COMP-G*).

When we applied the decomposition (Fig. 1) to our data, we found that low reef fish biomass between reference and comparison sites was associated predominantly with changes in richness and composition rather than environmental and other drivers (Fig. 3). The cumulative biodiversity effect *DIV* was 5.7x stronger (95% confidence intervals: [5.1, 6.5]) than the *CDE* effect (Fig. 3). Furthermore, the biodiversity effect on community biomass was driven almost entirely by the loss (*L*) rather than the gain (*G*) of species (Fig. 3). We note that this finding is not an artifact of the partitioning equation: indeed, high-biomass reference sites are not mathematically constrained to have more species, but apparently do in nature. The majority (69%) of comparisons to the reference site exhibited a net loss rather than a net gain of species (−11.4 ± 0.7% average (± SE) reduction in the number of species across all comparisons). Thus, our analysis indicates that differences in biodiversity, primarily those that reflect the absences of species from site to site, are the primary determinants of fish community biomass across the world’s coral and rocky reefs over the scale of 10 s of kilometers.

The strongest effect of biodiversity loss on community biomass was due to changes in species composition. Loss of biomass attributable to changing species identities (*COMP-L*) was 3.8x greater (95% CIs: [3.5, 4.1]) than that based on the “expected” or average outcome of removing species at random (*RICH-L*, Fig. 3). In approximately three-quarters of cases, the *COMP-L* term was negative, indicating that compositional effects were primarily due to the loss of greater-than-average contributors to biomass (i.e., large-bodied fishes) and/or those with extreme abundances (e.g., schooling fishes). Thus, we demonstrate that species’ identities and their traits (e.g., body size, gregariousness) are key factors in predicting the ecosystem consequences of species’ losses. We note that the strong effect of species identity does not negate the contributions of richness: indeed, reductions in the number of species necessarily change composition, and thus both operate simultaneously to influence community biomass.

Our finding that biodiversity loss is the primary determinant of fish community biomass among reefs within a region is robust to the spatial extent over which the local comparisons are conducted (15-–100 km, Supplementary Fig. 1), with slightly weaker effects of composition observed at reduced scales (mean *COMP-L* = −0.38, −0.38, −0.40, and −0.43 for 15-, 25-, 50- and 100-km radii, respectively). The proportion of species shared among any two sites, however, remained remarkably consistent from scales of 15–100 km (49.1–51.5% of the total community), and thus our results do not appear to be biased by an increased overlap in composition between communities at smaller scales or by more rare or unique species at larger scales. Additionally, our results are largely consistent across temperate and tropical realms (Supplementary Fig. 2), with two notable exceptions. First, we observed slightly more extreme declines in biomass due to richness losses in tropical areas, presumably because tropical sites have, on average, 2.8x more species than temperate ones and therefore possess more scope for change. Second, we observed similarly larger declines in biomass due to compositional losses at temperate sites, reflecting that individuals there are, on average, 1.5x larger than at tropical sites and therefore more likely to contribute biomass in excess of the “expected” or average species. Nevertheless, these two differences cancel each other out such that the total diversity effect (*DIV*) does not differ between the two realms (Supplementary Fig. 2).

**Fig. 4:**
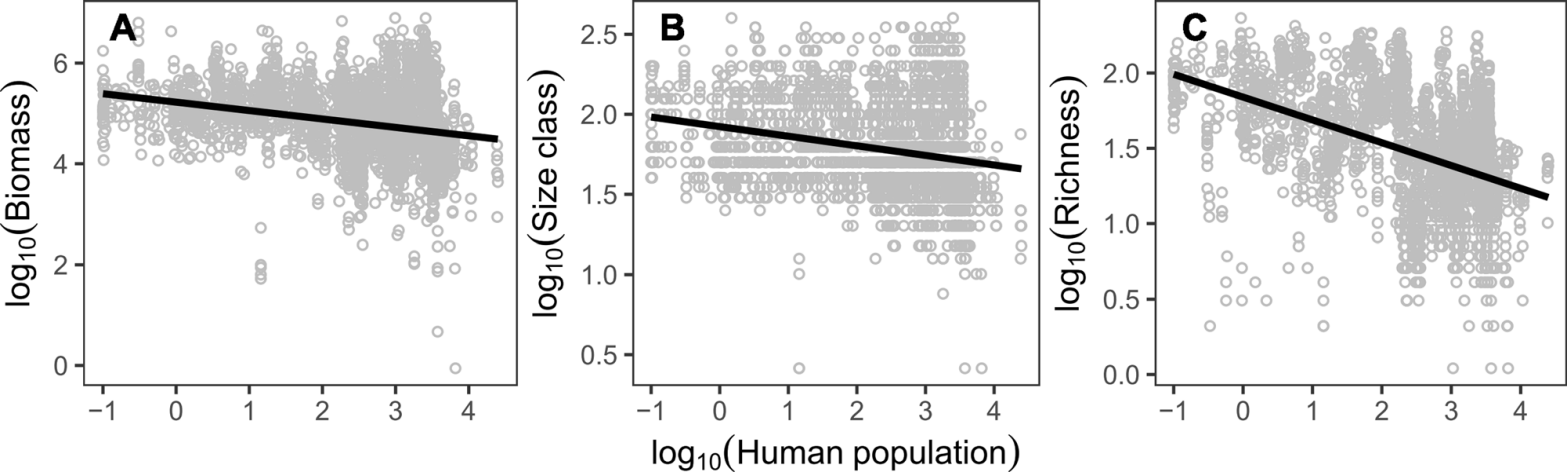
Human population size predicts aspects of fish community structure. Proximity to high densities of humans is associated with: **A** reduced total fish community biomass; **B** smaller observed median size classes; and **C** fewer species. Lines are predicted trends from generalized linear models.

To understand the potential drivers of our results, we used random forest analysis to identify the environmental and anthropogenic variables most associated with each component of our decomposition^18^. The top-ranked predictor for biomass loss associated with richness and compositional loss was consistently human population density, particularly for the *COMP-L* and *DIV* terms (Supplementary Fig. 3). Sites near even a minimal human population exhibited lower total fish biomass, smaller-bodied fishes, and fewer fish species (*P* < 0.001 in all cases, from generalized linear mixed-effects models regressing fish community characteristics against human density) (Fig. 4). Our index provides a proxy for a range of human impacts, including direct removal of species and biomass through fishing and the indirect effects of numerous factors such as reduction in suitable habitat and food availability^9,19^. In contrast, the context-dependent effect (*CDE*) was most influenced by environmental variables, specifically salinity, nutrients (dissolved phosphorus), and temperature variation (Supplementary Fig. 3). This result would be expected when environmental variation affects the weight and abundance of individuals but does not exclude species entirely, clarifying our interpretation of the *CDE* term as capturing underlying gradients that might alter the capacity of species to acquire resources and produce biomass. Environmental factors also affected the diversity terms (e.g., phosphorus for *RICH-L*, Supplementary Fig. 3), albeit to a lesser degree, reflecting that environment can also be responsible for driving local species absences or latently indicate a degraded state which certain species find unsuitable.

**Fig. 5:**
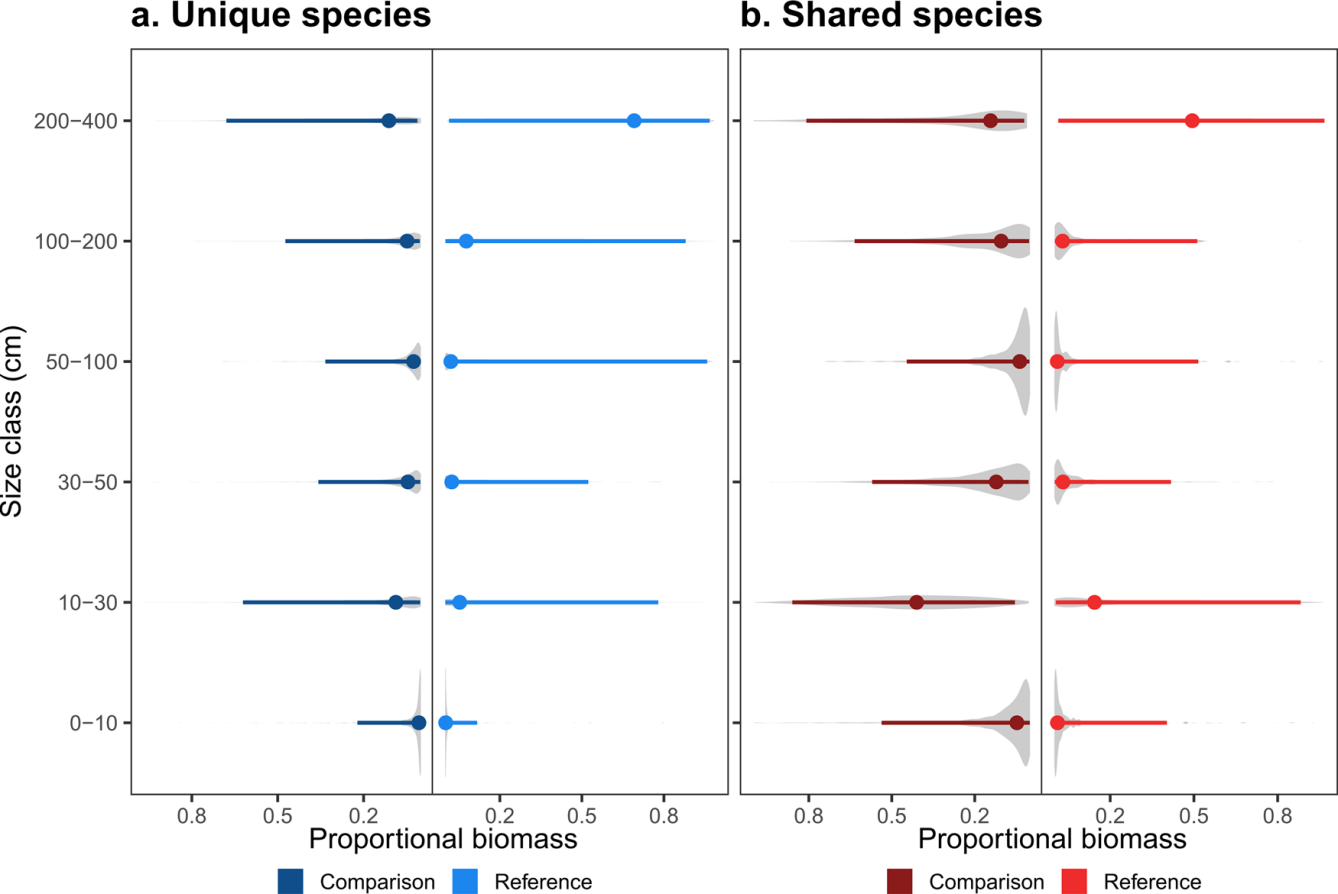
Contributions of fish size to total fish biomass at comparison and reference sites. **A** A much larger proportion of the biomass of species unique to reference sites was attributable to large fishes (>200 cm), as compared to species that were only found at comparison sites. **B** Among those species that were shared between both the reference and comparison sites, small fish (<50 cm) made up a much larger proportion of the total biomass, especially at the comparison site. Points are medians ±95% quantiles, and the gray shaded area shows the underlying distribution of raw data. The total number of individuals represented in the plot is *n* = 41,267.

The effects of species identity on community biomass recovered by our analysis are most likely related to selective removal of large species, as found in prior studies of fisheries^20,21^, rather than changes in the number of individuals. To test this hypothesis, we partitioned the contributions of species unique to reference and comparison sites as a function of size class. As expected, the unique contributors at the most productive reference sites were concentrated in the largest size class (>200 cm observed total length) (Fig. 5). Of species unique to the reference sites, 56.4% of the biomass was contributed by fishes >200 cm in length, whereas this size class contributed only 19.0% of the total biomass at the comparison sites. These observations also explain the bimodal distribution of the *COMP-L* term: the majority of highly negative values reflect losses of larger-than-average species from reference sites, as opposed to smaller-than-average fishes which are captured in the minority of positive values of *COMP-L* (Fig. 3). The lack of large species at comparison sites could have resulted from the inclusion of species that are more mobile and have larger home range sizes, and just so happen to have occurred at a particular reference site. To test whether the contributions of such species might have unduly influenced our results, we re-ran our analysis after removing all 156 pelagic/non-site attached species from our dataset (~5% of all species, classifications from^22^). This subsequent analysis yielded nearly identical results to those from the full dataset (Supplementary Fig. 4).

We set the reference site as the highest biomass site to simplify the interpretation of biomass variation across communities because this makes the net change always negative^13^. Analyzing the data in this way allows us to ask whether losses in biomass are due primarily to cumulative changes in diversity, broadly defined (*DIV*), or losses within species (*CDE*). However, this choice of reference may also bias towards a compositional effect, since larger species that contribute most to biomass are more likely to be found at high-biomass sites. To test for this bias, we repeated our analysis on 1000 simulated versions of our original dataset that randomized composition while holding species numbers and per capita contributions constant. The value of *COMP-L* in the observed data was always more negative than those returned by our simulations *(P* < 0.001 based on a one-tailed comparison of means; Supplementary Fig. 5). Thus, our results do not appear to be an artifact of how the reference community was defined (see also Supplementary Materials for outcomes when alternatively establishing the reference as the most speciose sites). Instead, our findings reflect the inherent organization of natural systems: high-biomass sites support large-bodied species that are not often found elsewhere^23^.

Hundreds of experimental manipulations show that biodiversity loss is among the pre-eminent factors contributing to variation in community biomass in a wide range of ecosystem types and taxa driven by both changes in richness and composition^16,24^. Our study extends theoretical inferences from this work to highly diverse, natural ecosystems across a range of contexts. A synthesis of >200 terrestrial plant manipulative experiments revealed roughly equivalent contributions by species richness and species identity^10^, a result which matches our findings for global reef fishes. Here, we also provide a new mathematical approach to address this question. It is important to note that, like other recent ecological decompositions, ours is not a mechanistic model^12^. Instead, it integrates across all potential mechanisms driving the aggregate loss and gain of species, including their response to abiotic drivers, and how they affect ecosystem functioning. Therefore, our application permits disentangling richness vs. compositional effects operating in these communities^25^, but how these effects mechanistically arise (e.g., through dominance, complementary resource use, indirect interactions such as facilitation, etc.) requires further investigation^26^. Similarly, assessing processes such as production (for which we substitute standing stock biomass as a proxy) using this new partitioning is necessary if the goal is to link diversity to more robust measures of ecosystem functioning.

Our results strengthen previous findings that human actions have large impacts on fish biomass: many studies have demonstrated that humans are selectively removing large-bodied fishes from the ocean, largely on the basis of fisheries catch data^9,18,20,21,27,28^, resulting in a loss of functional diversity and disruption of ecosystem trophic structure^29^. Our study confirms a concerning ecosystem-scale consequence of this phenomenon: reductions in large-bodied species leads to greater divergence between high- and low-biomass sites. Our data, therefore, provide compelling new evidence that selective removal of species with particular traits and reduction of local biodiversity across the seascape through collective human impacts profoundly alter the structure and function of whole reef communities worldwide. This result has important implications for the continued management of reef ecosystems: conservation practices that are tied to the restoration of high-performing species^30^ and those that aim to preserve the diversity of whole communities (e.g., marine protected areas)^31,32^ are both required to maximize the provision of a critical ecosystem function in an increasingly human-dominated world^33^.

## Methods

### Reef life survey

Reef fish communities were censused by a combination of experienced marine scientists and trained recreational SCUBA divers using globally standardized Reef Life Survey methods. All surveys were undertaken on 50 m long transects laid along a contour (at consistent depth) on predominantly hard substrate (usually rocky or coral reef) in shallow waters (depth range of transects 1 to 20 m, average ~7.2 m). Full details of fish census methods, data quality, and training of divers are provided in refs. ^22,34,35^ and in an online methods manual (www.reeflifesurvey.com). Fish abundance counts and size estimates per 500 m^2^ transect area (2 ×250 m^2^ blocks) were converted to biomass using length–weight relationships for each species obtained from Fishbase (www.fishbase.org). In cases where length–weight relationships were provided in Fishbase using standard length or fork length, rather than total length as estimated by divers, length–length relationships provided in Fishbase allowed conversion to the total length. For improved accuracy in biomass assessments, observed sizes were also adjusted to account for the bias in divers’ perception of fish size underwater using an empirical calibration^36^. Length–weight coefficients from similar-shaped close relatives were used for those species where length–weight relationships were not available in Fishbase. All transects were collapsed into a single average value of biomass for each species at a location to account for any differences in the total number of transect surveys performed.

### Decomposition of difference in ecosystem functioning

Our equation was inspired by previous decompositions, principally the Price equation originally derived in the field of evolutionary biology as a means of separating genetic and environmental influences on phenotypic change over time^37^. Fox^38^ and later Fox and Kerr^12^ modified the Price equation to describe how the difference in the ecological function between two communities can be decomposed into components with different ecological interpretations. We follow a similar approach but use a different decomposition where the resulting components are similar to, but not the same as, the components proposed by Fox and Kerr^12^.

We begin by assuming that the ecological function of the community, such as biomass, is a simple additive function of the contributions of its constituent species. We go on to compare two communities, one of which we consider the “reference” community and the other we refer to as the “comparison” community. The species present in the reference community can be classified into two types: species that are unique to the reference community (i.e., not present in the comparison community) and those that are in common with the comparison community. Let *s*_*uB*_ be the number of unique species in the reference community, and *s*_*c*_ be the number in common between the two communities. Let ${\bar{z}}_{{uB}}$ be the average ecological function contributed per unique species to the reference community, and ${\bar{z}}_{{cB}}$ be the average ecological function contributed per shared species in the reference community. The total ecological function *T*_*B*_ of the reference community can thus be decomposed as:

1 $${T}_{B}={s}_{{uB}}{\bar{z}}_{{uB}}+{s}_{c}{\bar{z}}_{{cB}}$$

where the first term represents the ecological function contributed by species that are unique to the reference community (i.e., not present in the comparison community) and the latter term represents the contribution from species that are also found in the comparison community.

Analogously, in the comparison community, the total ecological function can be decomposed as:

2 $${T}_{F}={s}_{{uF}}{\bar{z}}_{{uF}}+{s}_{c}{\bar{z}}_{{cF}}$$

with a similar interpretation to Eq. (1). Though there are *s*_*c*_ species in common between the two communities, the average per species contribution need not be the same in the two communities (i.e., ${\bar{z}}_{{cB}}$ may differ from ${\bar{z}}_{{cF}}$).

The species in common between the two communities can serve as a reference point for comparison between communities. It is useful to define ${\delta }_{B}={\bar{z}}_{{uB}}-{\bar{z}}_{{cB}}$ and ${\delta }_{F}={\bar{z}}_{{uF}}-{\bar{z}}_{{cF}}$ as the difference in average ecological function per species of unique species versus shared species in reference and comparison communities, respectively. From this perspective, we consider the average ecological function of a species unique to the reference community as being equal to the average ecological function of shared species (as measured in the same community) plus the deviation from this value ${\bar{z}}_{{uB}}={\bar{z}}_{{cB}}+{\delta }_{B}$. Using this equality and the analogous one for ${\bar{z}}_{{uF}}$, along with Eqs. (1) and (2), the difference in the ecological function between communities can be decomposed as

3 $$\Delta T={T}_{F}-{T}_{B}={-s}_{{uB}}{\bar{z}}_{{cB}}-{s}_{{uB}}{\delta }_{B}+{s}_{{uF}}{\bar{z}}_{{cF}}+{s}_{{uF}}{\delta }_{F}+{s}_{c}\left({\bar{z}}_{{cF}}-{\bar{z}}_{{cB}}\right)$$

The first two terms represent the loss in ecological function in the comparison community due to the loss of species that are unique to the reference community. Specifically, the first term represents the loss in ecological function due to the absence of unique species if these species had the same average value of functioning as each of the shared species. In other words, it is the amount by which biomass is expected to decline if species were interchangeable. Therefore, we interpret this term as the “richness loss” or the loss in functioning due strictly to the loss of species: *RICH-L* ($={-s}_{{uB}}{\bar{z}}_{{cB}}$). It will always be negative, assuming there is at least one species unique to the reference population. In cases where ${\bar{z}}_{{cB}} > {\bar{z}}_{{uB}}$, it is possible for *RICH-L* to exceed the total functioning observed at the reference site, which complicates interpretation of the raw values. In this case, it is useful to consider only the relative quantities (each component is scaled by the sum of the absolute values of all components). We note that this situation arises only 41 times out of 2867 comparisons in our analysis, and removing these cases has no effect on our findings. We advise future applications be aware of this potential issue and test for its influence.

The second term accounts for the fact that the true loss in ecological function due to these lost species will often differ from the “richness expectation” because the lost species differ in value from the average value of shared species. In other words, this term reflects the deviation in the actual contributions of lost species from the average of shared species, which implies that not all species contribute equally (and that the identities of the species are important in determining differences in biomass between the two communities). We, therefore, interpret this term as indicating “compositional loss,” or the degree to which loss in biomass is due to loss of particular species: *COMP-L* ($= - {s}_{{uB}}{\delta}_{B}$). If the average lost species provide a higher contribution to the reference community than the average shared species (${\bar{z}}_{{uB}} > {\bar{z}}_{{cB}}$), the *COMP-L* term will be negative. On the other hand, if the average lost species represent lower contributions, the *COMP-L* term will be positive (${\bar{z}}_{{uB}} < {\bar{z}}_{{cB}}$).

The next two terms are analogous to the first two terms but instead represent the increase in ecological function in the comparison community due to the “gain” of unique species that are lacking from the reference community. The third term represents the expected increase in ecological function due to an increase in species richness assuming these gained species had the same per species contribution as the shared species: *RICH-G* ($={+s}_{{uF}}{\bar{z}}_{{cF}}$). It is always positive, assuming the comparison community has at least one unique species. The fourth term, *COMP-G* ($=+{s}_{{uF}}{\delta }_{F}$), reflects the difference in composition (with respect to average value) of gained versus shared species. This term can be positive or negative, being positive if the gained species have a higher per species value than the shared species.

The final term focuses on the changes in biomass considering only the species that are present in both communities. This can be thought of as holding richness and composition constant and considering changes in the community biomass that are controlled extrinsically, i.e., by underlying gradients in resource availability and other environmental factors. Historically, this term has been referred to as the “context-dependent effect,” or *CDE*, and is the number of shared species (${s}_{c}$), multiplied by the difference in biomasses among shared species at both sites ($={s}_{c}({\bar{z}}_{{cF}}-{\bar{z}}_{{cB}})$). It can be of either sign: positive if shared species have a higher value in the comparison community than in the reference, negative if they have a higher value in the reference community. The number of shared species has the potential to bias away from the *CDE* term if it is very low. However, we note that, on average, 49.1 ± 0.003% of species are shared for each comparison at the 100-km scale, and this value is remarkably consistent regardless of spatial scale (51.3–50.0% for 15–50 km).

Our decomposition is similar to, but not the same as, that of Fox and Kerr^12^, though both are mathematically sound. Only the *CDE* term is mathematically identical across the two decompositions and, thus, shares the same interpretation. By extension, the sum across the loss and gain terms (the total diversity effect, or *DIV*) must also be identical, because both equations partition the same total quantity. Thus, it is important to note that using either decomposition yields the same inference with respect to comparisons of *DIV* and *CDE*.

Our decomposition differs from Fox and Kerr’s because the two approaches use different reference points. We take the perspective that the shared species form the basis for comparison between two communities, so we then evaluate the average value of a unique species with respect to its deviation from an average value of a shared species. In contrast, Fox and Kerr effectively evaluate the average value of a unique species with respect to its deviation from the average value of any species in that community (averaging over both unique and shared species). In both decompositions, the “composition” components only exist if there is some difference in the average value of shared and unique species. We prefer our decomposition for this case because it works with that difference directly rather than indirectly via the difference between unique and all species (which is the average of unique and shared species). Moreover, our composition makes intuitive sense that the function of the “average” species is determined by the ones that are known to exist at both sites. A full comparison of the Fox and Kerr formulation and ours is provided in the Supplementary Materials.

### Statistical analysis

A general function to conduct our new decomposition from a site-by-species biomass matrix, and a second function to perform the simulations, can be found here: https://gist.github.com/jslefche/76c076c1c7c5d200e5cb87113cdb9fb4.

We first ordered all sites by decreasing total biomass. Beginning with the highest biomass site of all sites as the first reference site, we identified all other sites within a certain spatial radius (15-, 25-, 50-, or 100-km) to serve as the comparison sites. Setting the reference to be the site with the highest community biomass constrains the sum of the terms to be negative. This choice simplifies the language used to discuss the output^13^ and allows us to speak directly to the consequences of real-world activities like overharvesting (and their implications).

We then computed the components for each set of comparisons. We standardized the output to the same scale (−1, 1) by first taking the sum of the absolute value of all components, and then dividing each component by this value. This relativization was done to account for the fact that raw biomass may differ substantially among sites and regions and to make our results comparable across the entire dataset. Once the scaled components were computed, the reference and comparison sites were removed from the ordered list from any further comparisons to prevent any bias that might arise from including the same site multiple times. We then moved onto the next most productive site in the list, identified the comparison sites within 100 km, computed the components, and so on, until all sites were analyzed. From these individual comparisons, we computed the means of all components while omitting any reference sites for which there were fewer than five comparison sites. We alternately averaged the components for all comparisons for each reference site and then took the grand mean of these averaged values, although this additional level of aggregation did not qualitatively change our results (Supplementary Fig. 6). We have chosen to present the raw values in the main text to demonstrate the full range of variability inherent in the individual comparisons, which might otherwise be condensed by showing only the means for each reference site. We repeated the analysis over multiple spatial radii to assess whether the spatial extent and therefore the size and composition of the species pool, might influence our results.

We calculated the relative strength of the total diversity effect vs. the context-dependent effect for each comparison as the ratio of *DIV/CDE*, and of compositional vs. richness losses as:

4 $${{{{{\rm{Q}}}}}}=\frac{(-{s}_{{uB}}{\delta }_{B}{-s}_{{uB}}{\bar{z}}_{{cB}})}{{-s}_{{uB}}{\bar{z}}_{{cB}}}=\frac{{\bar{z}}_{{uB}}}{{\bar{z}}_{{cB}}}$$

In this case, *Q* = (*COMP-L* + *RICH-L*)/*RICH-L*, which reduces to the average value of unique species relative to the average value of shared species at the reference site. This quantity reflects the magnitude to which species unique to the reference site contribute to biomass relative to the “expected” contribution per species. To avoid biases associated with averaging ratios, we report the geometric mean of both quantities. Bootstrapped 95% confidence intervals were derived by randomly resampling DIV/CDE and Q for a total of 5000 times. For DIV/CDE, some values were negative, so we excluded them in both the original data and bootstrap samples. As an alternative approach that focused on the magnitude of effect, we examined the absolute value of |DIV | / | CDE | . In this case, the ratio was 6.9x with bootstrap 95% CIs of [6.2, 7.7].

To explore the drivers of the components of our decomposition, we applied random forest analysis to account for potential collinearity and interactions among the suite of predictors previously selected in ref. ^39^. Depth was recorded on the surveys while the following predictors were obtained from the combination of remote sensed and in situ measurements compiled in the Bio-ORACLE database: mean, minimum, maximum, and range of sea surface temperature; mean, minimum and maximum for surface chlorophyll-*a*; mean salinity; mean PAR; mean dissolved oxygen; mean nitrate concentration; mean phosphate concentration^40^. Finally, an index of human population density was calculated by fitting a smoothly tapered surface to each settlement point on the year 2010 world-population density grid using a quadratic kernel function described previously^41^. Random forests were fit using the default settings in the *randomForest* package^42^ in R version 4.1.1^43^. Variable importance was determined using the percent increase in the mean-square error after randomly permuting the predictor of interest for each tree in the random forest, averaging the error of the models, and then computing the difference relative to the accuracy of the original model.

### Null simulations

A key finding of our analysis is that compositional losses are considerably greater than losses due to other aspects of the reef fish community. We wanted to evaluate the possibility of whether such a result could be an artifact of applying our decomposition to a dataset in which we assign the site with the higher total biomass as the “reference” community and the site with lower total biomass as the “focal” community. To do so, we conducted simulations in which we created communities with species richness values matching the observed data, but for community compositions that were random. Following the same procedure we used with the real communities, we applied our decomposition to these simulated communities to generate null distributions for the average values of each of the five terms when community composition is random. Comparing our observed values to these null distributions tells us if the values of the compositional components (or indeed any component) we observed arose as an artifact of our procedure or, alternatively, because high-biomass sites actually contain more high-biomass species than expected under random community assembly.

Our simulation procedure focused on the site-by-species biomass matrix from each set of comparisons used in the main 100-km analysis. We divided this matrix by the corresponding site-by-species abundance matrix to yield the observed per capita contribution of each species in each community. We then averaged the per capita contributions of each species across all communities where the species was present to yield a single vector representing mean per capita contributions for all *S* species within that set of comparisons.

We initially constructed each simulated community by populating it with every species in the region (“maximum richness”). To determine the biomass of each species in each community we applied the following procedure. First, we identified the minimum and maximum observed abundance of each species across all communities where it is present. For a single community, we sampled an integer value between the minimum and maximum abundance for each species to yield a single vector of random abundance values of length *S*, and then multiplied this vector by the vector of average per capita contributions. This procedure yielded a new vector representing a new total contribution to biomass by every species. We repeated this for all *n* communities in the original site-by-species matrix and bound these vectors together in a new “maximum richness” version of the site-by-species matrix. For the *i*th row (community) in the original dataset, we calculated the richness, *s*_*i*_. We then randomly subsampled *s*_*i*_ species at random from the simulated “maximum richness” site-by-species matrix and set the biomass of any remaining species to zero. We repeated this for each community to yield a simulated “observed richness” site-by-species matrix with the same dimensions as the original matrix. This procedure ensures that richness is held at the observed levels and that the biomass contribution of each species are within the observed range.

These communities were intentionally constructed randomly with respect to composition as our goal was to test whether the observed compositional effects in the real data are significantly different than under this null hypothesis with respect to composition. Thus, using the simulated “observed richness” site-by-species matrix, we computed the (scaled) components as we had with the real data and took their means across all communities. We repeated the randomization procedure 1000 times to yield 1000 total average values of each component. We compared the observed mean to the distribution of expected means using a one-tailed *t-*test to determine whether the observed components were more or less extreme than would be expected by chance.

### Reporting Summary

Further information on research design is available in the Nature Research Reporting Summary linked to this article.

## Supplementary information

Supplementary Materials

Peer Review File

Reporting Summary

## Data Availability

The data generated in this study have been deposited in the figshare database at: 10.25573/serc.16847029.

## Code availability

The code to reproduce all analyses has been deposited in the figshare database at: 10.25573/serc.16847029.
