# Publisher Correction: Species richness and identity both determine the biomass of global reef fish communities

**DOI:** 10.1038/s41467-021-27681-y

**Published:** 2021-12-16

**Authors:** Jonathan S. Lefcheck, Graham J. Edgar, Rick D. Stuart-Smith, Amanda E. Bates, Conor Waldock, Simon J. Brandl, Stuart Kininmonth, Scott D. Ling, J. Emmett Duffy, Douglas B. Rasher, Aneil F. Agrawal

**Affiliations:** 1 Tennenbaum Marine Observatories Network and MarineGEO Program, Smithsonian Environmental Research Center, Edgewater, MD 21037 USA; 2 Institute for Marine and Antarctic Studies, University of Tasmania, Hobart, TAS 7001 Australia; 3 Department of Ocean Sciences, Memorial University of Newfoundland, St. John’s, NF A1C 5S7 Canada; 4 Department of Biology, University of Victoria, Victoria, BC V8P 5C Canada; 5 Landscape Ecology, Institute of Terrestrial Ecosystems, ETH Zürich, CH-8092 Zürich, Switzerland; 6 Aquatic Ecology and Evolution, Institute of Ecology and Evolution, University of Bern, Bern, Switzerland; 7 Department of Marine Science, The University of Texas at Austin, Marine Science Institute, Port Aransas, TX 78373 USA; 8 School of Marine Studies, The University of South Pacific, Laucala Bay Road, Suva, Fiji Islands; 9 Bigelow Laboratory for Ocean Sciences, East Boothbay, ME 04544 USA; 10 Department of Ecology & Evolutionary Biology, University of Toronto, Toronto, ON M5S 3B2 Canada

**Keywords:** Community ecology, Ocean sciences, Biodiversity

Correction to: *Nature Communications* 10.1038/s41467-021-27212-9, published online 25 November 2021.

In this article the caption to Fig. 2 was inadvertently truncated. The original article has been corrected.

