# Supplementary material for: Species richness and identity both determine the biomass of global reef fish communities
Source: Nat Commun. 2021 Nov 25;12:6875. doi: 10.1038/s41467-021-27212-9 (PMC8616921; doi:10.1038/s41467-021-27212-9)
Supplement: Supplementary file 1 — Supplementary Materials [file 41467_2021_27212_MOESM1_ESM.pdf]

Supplementary Information File

Species richness and identity both determine the biomass of global reef fish communities

Jonathan S. Lefcheck, Graham J. Edgar, Rick D. Stuart-Smith, Amanda E. Bates, Conor Waldock, Simon J. Brandl, Stuart Kininmonth, Scott D. Ling, J. Emmett Duffy, Douglas B. Rasher, Aneil F. Agrawal

Supplementary Discussion

Choice of an alternate reference

To test the effect of reference choice on our results, we alternately defined the reference as the most speciose sites. This choice asks whether removing species is more associated with losses or gains in community biomass, which is perhaps more analogous to the typical biodiversity-function experiment [23]. When we repeated our analysis but set the reference sites as those with the most species, we found that biodiversity loss was still the most important factor in determining community biomass (Supplementary Fig. 7). However, the loss of species tended to reduce biomass as much as compositional losses. In other words, more speciose sites did not harbor unique contributors to biomass to the same degree as high-biomass sites in our original analysis (Fig. 5), which is to be expected as most species are small (median observed size class = 12.5 cm TL). This reference also reflects a less conservative test of our hypothesis. Previously, it was possible to remove biomass without changing diversity (e.g., through the *CDE* term), while the inverse is not true: removing species will necessarily reduce biomass, therefore biasing towards the *RICH-L* component and inflating its relative importance. Nevertheless, our inferences regarding the combined influence of diversity are sustained: the total diversity effect

(*DIV*) was 3.77x (95% confidence intervals: [3.45, 4.11]) more negative than the context-dependent effect (*CDE*) when using this reference.

Comparison of our decomposition with that of Fox and Kerr (2012)

There are numerous mathematically valid ways one could decompose the difference in biomass (or any aspect of ecological function) between two communities. Some decompositions will result in more easily interpretable or biologically meaningful terms than others. Among the many options, there is no single "perfect" choice, though some are better than others given the study objectives. Here, we review the derivation of various ecological decompositions and contrast them with the one we present in the current study.

Fox (2006) pioneered the idea of decomposing the difference in biomass (or any additive aspect of ecological function) between two communities into richness, compositional, and context-dependent effects. The decomposition of Fox (2006) only applies when the focal community has a strict subset of the species present in the reference community. Using the methodology of Kerr and Godfrey-Smith (2009), that assumption was relaxed by Fox and Kerr (2012), hereafter FK12. The FK12 decomposition was a key inspiration for our work but we found that an alternative decomposition did a better job of quantifying the biological ideas motivating our research. The two decompositions are related but not the same. In this section, we compare these two decompositions. We begin with a mathematical comparison but readers who are less mathematically inclined may choose to skip directly to the examples.

Mathematical comparison

When comparing two communities, there are three categories of species: (i) those unique to the baseline (or "reference") community; (ii) those unique to the focal (or "comparison")

community; and (iii) those in common between the two communities. The total biomass of the two communities (T_B and T_F, for baseline and focal communities respectively) as well as difference between them ($\Delta T = T_F - T_B$) depends on two simple properties for each category: the number of species in the category and the average biomass per species (represented in the table below).

Supplementary Table 1. Symbols for key model parameters.

**Category Of species**	**Number in *baseline* site**	**Average biomass per species in *baseline* site**	**Number in *focal* site**	**Average biomass per species in *focal* site**
Species *unique* to the **baseline** site	s_{uB}	$\bar{z}_{uB}$	NA	NA
Species *unique* to the **focal** site	NA	NA	s_{uF}	$\bar{z}_{uF}$
Species *common* to both sites	s_c	$\bar{z}_{cB}$	s_c	$\bar{z}_{cF}$
All species	$s_B = s_{uB} + s_c$	$\bar{z}_B = \frac{s_{uB}\bar{z}_{uB} + s_c\bar{z}_{cB}}{s_{uB} + s_c}$	$s_F = s_{uF} + s_c$	$\bar{z}_F = \frac{s_{uF}\bar{z}_{uF} + s_c\bar{z}_{cF}}{s_{uF} + s_c}$

As discussed in the main text, our decomposition is expressed in terms of these simple properties:

$$\Delta T = -s_{uB}\bar{z}_{cB} + s_{uF}\bar{z}_{cF} - s_{uB}(\bar{z}_{uB} - \bar{z}_{cB}) + s_{uF}(\bar{z}_{uF} - \bar{z}_{cF}) + s_c(\bar{z}_{cF} - \bar{z}_{cB}) \quad \text{[E1]}$$

The five terms correspond to RICH-L, RICH-G, COMP-L, COMP-G, and CDE, respectively.

The derivation for the FK12 decomposition was heavily inspired by the Price equation used in evolutionary biology. It was presented in the following form:

$$\Delta T = (s_c - s_B)\bar{z}_B + (s_c - s_F)\bar{z}_F + Sp(w_{.}^{I}, z) + \left[-Sp\left(w_{j}^{.}, z\right)\right] + \sum_{i=1}^{s_c} \Delta z_i \qquad \text{[E2]}$$

We have adjusted their notation slightly to make it more compatible with ours. Here $s_B = s_c + s_{uB}$ [total species number at the reference site (FK12 uses s)], $s_F = s_c + s_{uF}$ [total species number at the focal site (FK12 uses s')], $\bar{z}_B$ is the mean of *all* species at the reference site (FK12 uses $\bar{z}$), $\bar{z}_F$ is the mean of *all* species at the reference site (FK12 uses $\bar{z}'$), and $Sp(x,y)$ is the sum of products operator and FK12 use w values as indicators of whether a species is present at both sites (see their paper for details). The first two terms are what they refer to as their "richness" loss and gain terms. The next two terms are what they refer to as their "composition" loss and gain terms. The final term is their CDE.

With some additional algebra one can express all five terms of [E2] with respect to the simple properties in Supplementary Table 1. For interested readers, we show below how to do this for their compositional loss term; others may skip directly to equation [E3] and the subsequent text. The compositional loss term of FK12, $Sp(w_{.}^{I}, z)$, is more explicitly defined in Fox (2006) as:

$$Sp(w_{.}^{I}, z) = \sum_{i}^{s_B = s_c + s_{uB}} (w_i - \bar{w}_B)\left(z_{B,i} - \bar{z}_B\right)$$

where the summation is only over the species present in the baseline community. w_i is 1 if the species is shared between communities and 0 if it is not. Thus, $\bar{w}_B = s_c/(s_c + s_{uB})$ is fraction of species in the baseline community that are shared. It is useful to split right-hand side into two components, representing the shared species and the unique species and noting that $w = 1$ for the shared species and $w = 0$ for the unique species:

$$Sp(w_{\cdot}^{I}, z) = \sum_{i=1}^{s_c} \left(1 - \frac{s_c}{s_c + s_{uB}}\right)(z_{B,i} - \bar{z}_B) + \sum_{i=s_c+1}^{s_B=s_c+s_{uB}} \left(0 - \frac{s_c}{s_c + s_{uB}}\right)(z_{B,i} - \bar{z}_B)$$

$$= \frac{s_{uB}}{s_c + s_{uB}} \sum_{i=1}^{s_c} (z_{B,i} - \bar{z}_B) - \frac{s_c}{s_c + s_{uB}} \sum_{i=s_c+1}^{s_B=s_c+s_{uB}} (z_{B,i} - \bar{z}_B)$$

The two summations can now be expressed in terms of the mean of shared and unique species, respectively,

$$Sp(w_{\cdot}^{I}, z) = \frac{s_{uB}}{s_c + s_{uB}}(s_c\bar{z}_{cB} - s_c\bar{z}_B) - \frac{s_c}{s_c + s_{uB}}(s_{uB}\bar{z}_{uB} - s_{uB}\bar{z}_B)$$

$$= \frac{s_c s_{uB}}{s_c + s_{uB}}((\bar{z}_{cB} - \bar{z}_B) - (\bar{z}_{uB} - \bar{z}_B))$$

$$= \frac{s_c s_{uB}}{s_c + s_{uB}}(\bar{z}_{cB} - \bar{z}_{uB})$$

An analogous approach can be applied to their compositional gain term. The remaining terms are straightforward to express with respect to the simple properties in Table S1. Writing the terms in the same order as in equation [E2] above gives FK12 decomposition as

$$\Delta T = -s_{uB}\bar{z}_B + s_{uF}\bar{z}_F - \frac{s_c s_{uB}}{s_c + s_{uB}}(\bar{z}_{uB} - \bar{z}_{cB}) + \frac{s_c s_{uF}}{s_c + s_{uF}}(\bar{z}_{uF} - \bar{z}_{cF}) + s_c(\bar{z}_{cF} - \bar{z}_{cB})$$

[E3]

Both the FK12 and our decomposition have terms described as representing “richness” and “composition” effects, but the parallel terms are not mathematically equivalent and neither are their biological interpretations.

The table below provides a side-by-side comparison of the two decompositions, both expressed with respect to the same simple parameters of Supplementary Table 1.

Supplementary Table 2. Side-by-side comparison of the two decompositions.

Term type	FK12 decomposition	Our decomposition
RICH-L	$-s_{uB}\bar{z}_B$ $= -s_{uB}\left(\frac{s_c}{s_c + s_{uB}}\bar{z}_{cB} + \frac{s_{uB}}{s_c + s_{uB}}\bar{z}_{uB}\right)$	$-s_{uB}\bar{z}_{cB}$
RICH-G	$s_{uF}\bar{z}_F$ $= s_{uF}\left(\frac{s_c}{s_c + s_{uF}}\bar{z}_{cF} + \frac{s_{uF}}{s_c + s_{uF}}\bar{z}_{uF}\right)$	$s_{uF}\bar{z}_{cF}$
COMP-L	$-\frac{s_c}{s_c + s_{uB}}s_{uB}(\bar{z}_{uB} - \bar{z}_{cB})$	$-s_{uB}(\bar{z}_{uB} - \bar{z}_{cB})$
COMP-G	$\frac{s_c}{s_c + s_{uF}}s_{uF}(\bar{z}_{uF} - \bar{z}_{cF})$	$s_{uF}(\bar{z}_{uF} - \bar{z}_{cF})$
CDE	$s_c(\bar{z}_{cF} - \bar{z}_{cB})$	$s_c(\bar{z}_{cF} - \bar{z}_{cB})$

The CDE effect is the same in both decompositions. This means the sum of the remaining four terms (DIV = RICH-L + RICH-G + COMP-L + COMP-G) is also the same and, indeed, it is. However, the four individual terms differ between the decompositions. Our decomposition is premised on using the shared species as the frame of reference for “standard” species. (This choice in frame of reference is a key difference between the decompositions and is further discussed below.) The expected loss of biomass due to number of species that are absent from the focal site (RICH-L) depends on the number of those species (s_{uB}) and average biomass of the “standard” species $\bar{z}_{cB}$. Our compositional effect directly captures the way those species absent from the focal community are different from standard (i.e., shared) ones $(\bar{z}_{uB} - \bar{z}_{cB})$, weighted

by how many species are absent (s_{uB}) from the focal community. This seems a straightforward way to quantify the ideas of "richness" and "composition" effects.

Now, compare the FK12 COMP-L effect to ours. It is the same as ours but multiplied by the fraction of species at the baseline site that are shared with the focal site, $s_c/(s_c + s_{uB})$. For our purposes, this extra factor obscures the biological effect in which we are most interested. Even if unique species contributed much more biomass per species than did shared species (e.g., $\bar{z}_{uB} \gg \bar{z}_{cB}$), the COMP term in the FK12 decomposition would go towards zero if there were many more unique species than shared species (e.g., $s_{uB} \gg s_{cB}$). Instead, the biomass lost from the loss of species is attributed almost entirely to a richness effect, which seems misleading for our interests. For the current study, we want the COMP terms to capture the consequences of unique species being different from shared species (with respect to average biomass). In the FK12 decomposition, that type of effect is muddied by the relative numbers of unique and shared species due to the averaging over all species as their reference set.

A notable feature of our decomposition is that the ratio of the total effect of species loss relative to just the richness effect on species loss $Q =$ (RICH-L + COMP-L)/RICH-L $= \bar{z}_{uB}/\bar{z}_{cB}$ is independent of the number species, which seems sensible given both RICH-L and COMP-L terms are quantifying effects from the same species (number and type) absent from the focal site. In our decomposition, the ratio Q directly captures the importance of the compositional difference of the lost species relative to those that are shared, i.e., whether unique species are more or less valuable in terms of biomass contribution per species than are shared species, which is our central question. For the FK12 decomposition, this ratio is harder to interpret: (RICH-L + COMP-L)/RICH-L $= (s_c + s_{uB})\bar{z}_{uB}/(s_c\bar{z}_{cB} + s_{uB}\bar{z}_{uB}) = s_B\bar{z}_{uB}/\bar{z}_B$.

Six hypothetical examples

We next present a few examples to contrast the two decompositions in some simple scenarios:

Example 1: The baseline and focal communities have the same number of species (i.e., no difference in species richness) but differ in composition. Specifically, the baseline community has a unique species of high value (e.g., a population of a large apex predator) whereas the focal community has a unique species of only modest value (e.g., a population of a small predator); see Supplementary Table 3.

Supplementary Table 3. Biomass value of species in hypothetical example 1.

**Species**	**Value in baseline community, *z***	**Value in focal community, *z***
Small_Sp1	20	20
Small_Sp2	25	25
Small_Sp3	30	30
Med_Sp1	50	50
Med_Sp2	absent	60
Large_Sp1	300	absent
TOTAL (*T*)	425	185

The two decompositions are presented side-by-side in Supplementary Table 4.

In this example, the number of species in the two communities is the same (i.e., no difference in species richness). In our decomposition, the net richness effect (= RICH-L + RICH-G) is zero. In the FK12, the net richness effect is -48. In our decomposition, the net composition effect (= COMP-L + COMP-G) is -240, which is equal to the total difference in between the communities.

Supplementary Table 4. Decomposition of hypothetical Example 1.

Term label	FK12 decomposition	Our decomposition
RICH-L	-85	-31.25
RICH-G	+37	+31.25
COMP-L	-215	-268.75
COMP-G	23	+28.75
CDE	0	0
Total change (ΔT)	-240	-240

In other words, our decomposition attributes all of the difference to a composition difference. This is arguably the correct interpretation of the difference here. In contrast, in the FK12 decomposition, the net effect of composition effects is -192; they attribute only 192/240 = 80% of the biomass difference to composition. The remaining 20% is attributed to a richness effect, which is an undesirable outcome given that the two communities have exactly the same species richness.

Example 2: This example is the same as Example 1 but with a few more species in common. We will simply add the same three new species to each community and they have the same value in both places (values: 25, 25, 55). The two decompositions are given in Supplementary Table 5.

Remember that in Example 2 the total change is the same as in Example 1 because the underlying ecological difference is the same, i.e., the two communities differ by the same composition of species in both examples.

Supplementary Table 5. Decomposition of hypothetical Example 2.

Term label	FK12 decomposition	Our decomposition
RICH-L	-66.25	-32.86
RICH-G	+36.25	+32.86
COMP-L	-233.75	-267.14
COMP-G	23.75	+27.14
CDE	0	0
Total change (ΔT)	-240	-240

In our decomposition of Example 2, the net richness effect remains 0 and the net composition effect remains -240. In the FK12 decomposition, the net richness effect has shrunk in magnitude to -30 and the net composition effect has grown in magnitude to -210 (but still only 87.5% of the total effect), demonstrating that it is sensitive to the number of shared species. In both examples, the difference between the communities is, arguably, entirely one of species composition as species richness does not differ between communities in either example. Moreover, the difference in species composition is exactly the same in both examples. Yet, the magnitude of FK12's net composition effect differs between the examples. Example 1 illustrates that the FK12 decomposition qualitatively misrepresents the biology from the perspective of our research goals by assigning a non-zero richness effect. Example 2 illustrates that, in the FK12 decomposition, the magnitude of the difference in biomass attributed to differences in species composition between communities depends on the species that are shared, not just the species that differ between the communities.

It would be misleading to depict our decomposition as working in a perfectly sensible manner under all possible circumstances. Indeed, a quick look at the terms in our decomposition identifies some scenarios where our decomposition might perform strangely.

Example 3: As in the previous examples, there is no difference in species richness between communities, only composition. However, in this example, the unique species have a lower value per species than the shared species (Supplementary Table 6). The decompositions are given in Supplementary Table 7.

Supplementary Table 6. Biomass value of species in hypothetical example 1.

**Species**	**Value in baseline community, *z***	**Value in focal community, *z***
Sp1	20	20
Sp2	25	25
Sp3	30	30
Sp4	absent	5
Sp5	10	absent
TOTAL (T)	85	80

The net richness effect in our decomposition is zero. The net composition effect is -5, matching the total change. However, now the compositional loss term is positive (when we typically expect a "loss" term to be negative) and the compositional gain term is negative (when we typically expect a "gain" term to be positive). This is strange in one sense but, fortunately, it is very simple to understand why it occurs from the math (Supplementary Table 2); it is a result of the unique species having a lower value than the shared species (e.g., $\bar{z}_{uB} < \bar{z}_{cB}$).

Supplementary Table 7. Decomposition of hypothetical Example 3.

Term label	FK12 decomposition	Our decomposition
RICH-L	-21.25	-25
RICH-G	+20	+25
COMP-L	+11.25	+15
COMP-G	-15	-20
CDE	0	0
Total change (ΔT)	-5	-5

The compositional *loss* term is of opposite sign to what we expect for a "loss" because the lost species are of lower value than "expected". The FK12 decomposition also has the same qualitative outcome with respect to the signs of the composition terms. As shown in Table S2, this is because, in *both* decompositions, the sign of the COMP-L term is determined by the sign of $\bar{z}_{uB} - \bar{z}_{cB}$ and the sign of COMP-G by the sign of $\bar{z}_{uF} - \bar{z}_{cF}$. Moreover, as in the previous examples, the FK12 decomposition attributes part of the total biomass change to a richness effect even though the two communities do not differ in richness (i.e., the net richness effect is -1.25). It is worth noting that the decompositions are simply different from one another so even in a simple case where both perform in a qualitatively sensible way, they give different quantitative values.

Example 4: This is the example given in the Appendix of Fox (2006). The baseline community has four species with values 1, 2, 3, and 4 respectively. The focal community is missing the first two species and the values of the last two species are 2 and 6. The decompositions are in Supplementary Table 8.

Supplementary Table 8. Decomposition of hypothetical Example 4.

Term label	FK12 decomposition	Our decomposition
RICH-L	-5	-7
RICH-G	0	0
COMP-L	2	4
COMP-G	0	0
CDE	1	1
Total change (ΔT)	-2	-2

Qualitatively, the two decompositions are the same; both have a negative RICH-L term, positive COMP-L and CDE terms, and the other terms are zero. The terms differ quantitatively because the frame of reference for the decompositions are different.

<u>Example 5</u>: In this example we compare a baseline community with two different focal communities. Each focal community is lacking a single species relative to the baseline community but differs with respect to which species is absent. The communities are represented in Supplementary Table 9 and the decompositions of the community comparisons in Supplementary Table 10.

Both focal communities have lost a single species relative to the baseline. In the FK12 decomposition the RICH-L term has the same value (-162.5) for comparisons of each focal community to the baseline. Arguably, that is a sensible feature of the FK12 decomposition. In contrast, in our decomposition the RICH-L term differs in the comparisons of each focal community to the baseline, even though each focal community lacks one species.

Supplementary Table 9. Biomass value of species in hypothetical Example 5.

Species	Value in baseline community, *z*	Value in focal community 1, *z*	Value in focal community 2, *z*
Sp1	50	50	50
Sp2	100	100	100
Sp3	200	200	absent
Sp4	300	absent	300
TOTAL (*T*)	650	350	450

Supplementary Table 10. Decomposition of hypothetical Example 5.

Term label	FK12 decomposition		Our decomposition	
	Focal Community 1	Focal Community 2	Focal Community 1	Focal Community 2
RICH-L	-162.5	-162.5	-116.7	-150
RICH-G	0	0	0	0
COMP-L	-37.5	-137.5	-183.3	-50
COMP-G	0	0	0	0
CDE	0	0	0	0
Total change (ΔT)	-300	-200	-300	-200

This discrepancy between the FK12 and our decomposition again traces back to how a ‘standard’ species is defined, which sets the expected value for a species loss. In the FK12 decomposition, the value of a standard species is defined by the average value of all species present at the baseline site. Thus, the expected value for a species loss does not depend on the composition of

the focal community to which it is being compared. In our decomposition, the value of a standard species is defined by the average value of only the species in common between the focal and baseline communities. Because the two focal communities share different sets of species with the baseline community, the frame of reference for the decomposition changes in the comparison of each focal community to the baseline. For certain questions this could be an undesirable feature of our decomposition. For example, if one is comparing several focal sites with a single baseline site then the issue highlighted here could complicate the interpretation of variation among such comparisons. However, FK12 does not evade this type of issue entirely as illustrated in the next example.

Example 6: This is the same as the previous example except that now each of the focal communities has its own species that is not present in the others. The communities are represented in Supplementary Table 11 and the decompositions in Supplementary Table 12.

Supplementary Table 11. Biomass value of species in hypothetical Example 6.

**Species**	**Value in baseline community, *z***	**Value in focal community 1, *z***	**Value in focal community 2, *z***
Sp1	50	50	50
Sp2	100	100	100
Sp3	200	200	absent
Sp4	300	absent	300
Sp5	absent	50	absent
Sp6	absent	absent	150
TOTAL (*T*)	650	400	600

Supplementary Table 12. Decomposition of hypothetical Example 6.

Term label	FK12 decomposition		Our decomposition	
	Focal Community 1	Focal Community 2	Focal Community 1	Focal Community 2
RICH-L	-162.5	-162.5	-116.7	-150
RICH-G	+100	+150	+116.7	+150
COMP-L	-137.5	-37.5	-183.3	-50
COMP-G	-50	0	-66.7	0
CDE	0	0	0	0
Total change (ΔT)	-300	-200	-300	-50

In this example, the total number of species does not differ among the three communities. As in Examples 1 and 2, in our decomposition the net effect of species richness (RICH-L + RICH-G) is zero, accurately reflecting the constancy of species richness. This is not the case for either community comparison with the FK12 decomposition. However, what if we contrast the the decompositions of the two focal communities with respect to the issue discussed in Example 5? In our decomposition, both the RICH-L and RICH-G terms differ in the two comparisons for the reason previously discussed. While the FK12 decomposition results in the same RICH-L term for both community comparisons, it results in different RICH-G terms despite the fact that each focal communities has only one species that the baseline community does not. This is because the FK12 decomposition uses two 'standards.' It uses one standard for setting the expectation for species losses, which is determined by all species at the baseline site and, thus, will be the same regardless of the composition of the focal community. It uses a different standard for setting the

expectation for species gains, which is set by the average value of all species at a focal site and thus will differ when a baseline is compared to communities of different composition. (When CDE effects are present, our decomposition also uses a different standard for expected losses and gains. The expected values for losses and gains are both based on the value of shared species but, for losses, it is their average value in the baseline site whereas it is their average value in the focal site for gains.)

Philosophical comparison

Why are the two decompositions different? At one level, it is simply because FK12 did the math differently than we did; both are valid, but different, mathematical routes that have the same starting place but lead to different end points. FK12 were heavily inspired by the Price equation of evolutionary biology and the logic that underlie that route. While the idea of decomposing biomass differences was heavily inspired by their work, our approach to the decomposition was different. Our decomposition was driven by thinking about what we wanted the terms to represent biologically and making use of the simple properties that must underlie all of the differences (the s and $\bar{z}$ values of the three categories of species depicted in Table S1).

From our vantage point, the key issue is the frame of reference. To evaluate the “richness” component, it is necessary to have some basis for the “expected” change due to a species loss (or gain). In other words, what value is lost from losing a single “standard” species? In our parlance, FK12 implicitly chose to represent a “standard” species (or, more accurately, the mean of the distribution of standard species) as the mean of *all* species at a site. In contrast, we chose to define a “standard” species by the mean of the *shared* species between sites. In using the shared species as the frame of reference in our decomposition, we evaluate the average value of unique species with respect to its deviation from the average value of shared species. This

approach explicitly separates the contributions of shared and unique species such that the compositional effect reflects the way the composition of the unique species is different from the composition of the shared species.

The choice of the frame of reference is, to some extent, a philosophical decision. This choice has mathematical consequences that affect what the resulting terms reflect biologically (see Table S2 and subsequent discussion). The odd feature of the FK12 COMP-L term that we discussed in Examples 1 and 2 is really just a mathematical consequence of FK12 implicitly using all species as the basis for a standard species. This is what leads to their RICH and COMP terms in Examples 1 and 2 behaving in a fashion that is undesirable with respect to our goals in the main text. On the other hand, as Examples 5 and 6 illustrate, our decision to use shared species as the frame of reference means that the expected value for a species loss or gain can differ for different communities compared to the same baseline. FK12 is not affected by this issue with respect to species losses but is with respect to species gains.

One biological justification for our choice to use shared species as the frame of reference is that we *know* shared species can exist in either community because *they do exist* in both communities. Species that are unique to one location may not be capable of persisting at other location for a variety of reasons and, thus, may be “special” (i.e., not standard) in some sense. We do not claim this “biological justification” constitutes an unassailable rationale for using our decomposition for all purposes. Arguments for other ‘standards’ could be made. The FK12 decomposition stems from the idea that there is a pristine or ecologically complete habitat that serves as the baseline and all of the species in that community should serve as the standard. Under that view, the species that happen to be shared with other communities should not define

the standard. Clearly, the choice of the frame of reference depends on one's perspective and what one seeks to learn from the decomposition.

Ultimately, no decomposition is perfect and different decompositions may prove useful for different questions. As shown above, both FK12 and our decomposition can sometimes result in seemingly strange but mathematically valid outcomes. It is helpful to understand what a decomposition is doing mathematically because this may not always match with the reader's intuitive perception of the linguistic labels given to the terms. However, it is very useful to remember that the difference in biomass between communities depends on the few simple properties given in Supplementary Table 1. Rather than relying solely on the output of any particular decomposition, it can be very helpful to look at the values of these simple properties directly to help ensure one is interpreting the decomposition reasonably.

Supplementary Figures

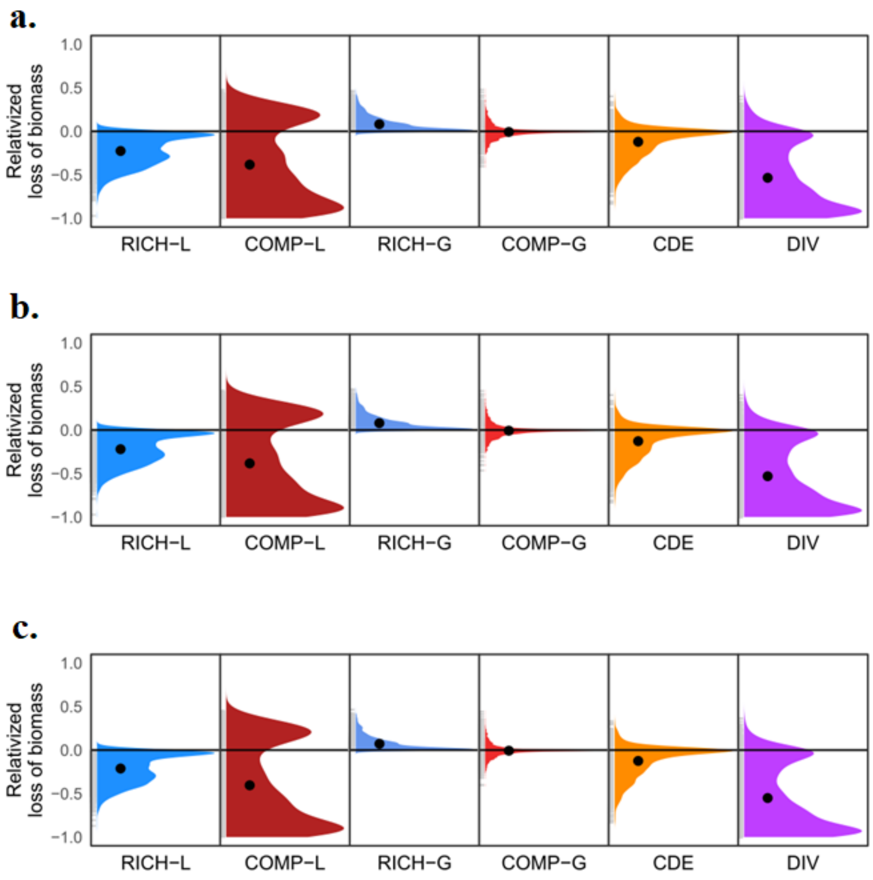

Supplementary Figure 1. Partitioning applied to comparison sites located within (a) 15-, (b) 25-, and (c) 50-km from the reference site. Interpretation is the same as Figure 2, main text.

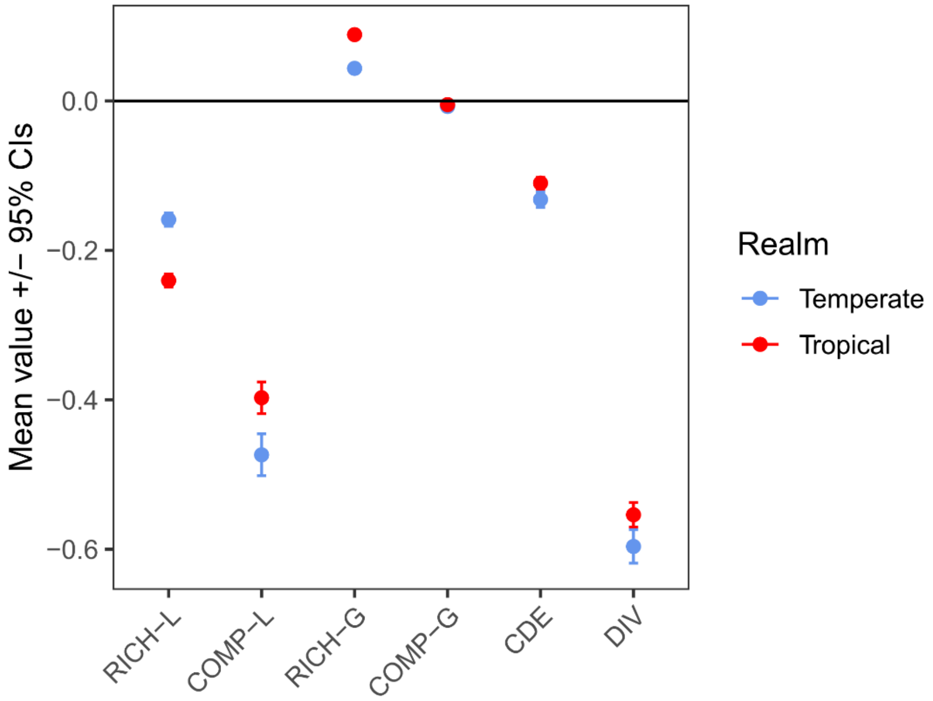

Supplementary Figure 2. Comparison of decomposition components across tropical (largely but not exclusively coral dominated) and temperate realms (predominantly rocky substrate). Points are means ± 1 standard error of the mean, and total number of comparisons n = 2867.

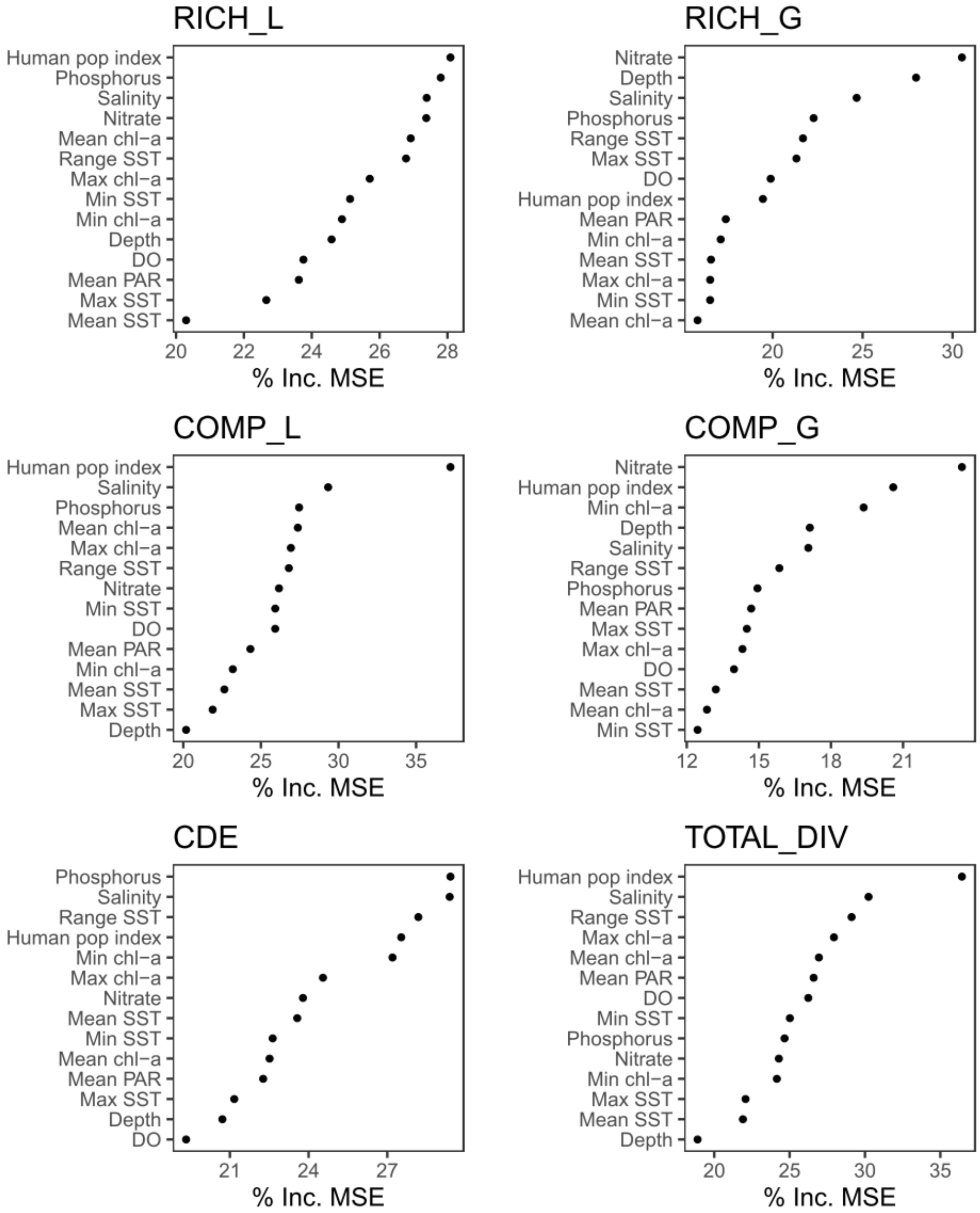

Supplementary Figure 3. Variable importance plots from individual random forests predicting each component of the modified decomposition of the Price equation. The x-axis is the percent increase in the mean-squared error after randomly permuting the variable of interest (y-axis): highly influential variables will therefore vastly increase the % MSE when their original values are changed.

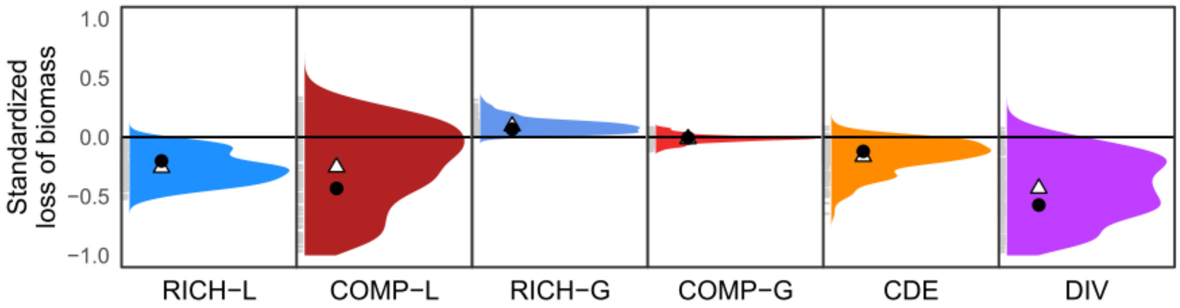

Supplementary Figure 4. Removal of large roaming pelagic species from the data (white triangles) revealed nearly identical inferences as the analysis on the full dataset (black circles), with a slightly diminished but still significantly negative *COMP-L* effect (as expected when removing larger-than-average species).

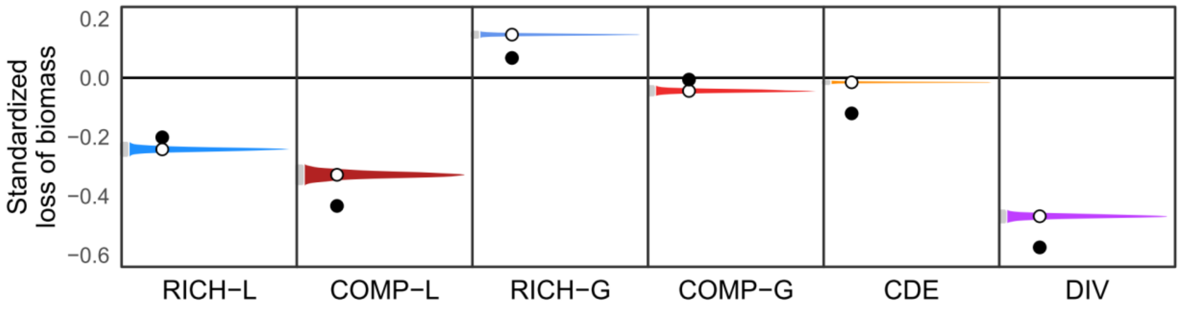

Supplementary Figure 5. Results from 1,000 random permutations of the observed community-by-species matrices while keeping richness constant. The distribution of the 1,000 simulated means for each of the components is given, as well as the grand simulated mean (white circles) and the observed mean from Figure 1, main text (black circles). In all cases, the observed means were more extreme than otherwise expected by chance.

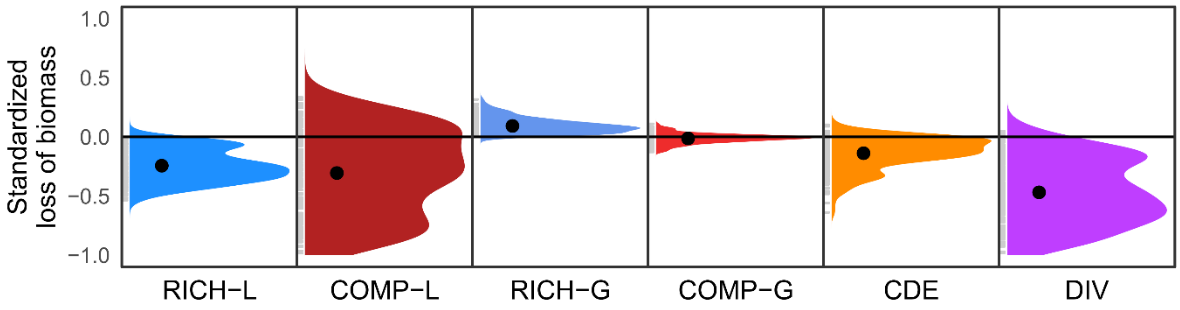

Supplementary Figure 6. Aggregating the data by each reference site (i.e., by taking the average value of each component across all comparisons for a given reference) revealed qualitatively identical results to the main analysis.

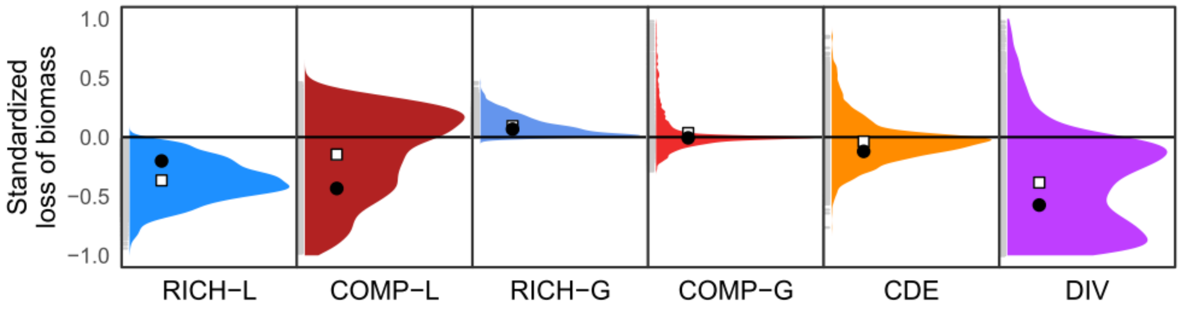

Supplementary Figure 7. Setting the reference as the most speciose site (white squares) generated similar relative inferences as setting the reference as the most productive site (black circles), although several components differed in absolute magnitude (e.g., *COMP-L*).
