# Supplementary material for: Species richness and identity both determine the biomass of global reef fish communities
Source: Nat Commun. 2021 Nov 25;12:6875. doi: 10.1038/s41467-021-27212-9 (PMC8616921; doi:10.1038/s41467-021-27212-9)
Supplement: Supplementary file 2 — Peer Review File [file 41467_2021_27212_MOESM2_ESM.pdf]

nature portfolio

Peer Review File

Species richness and identity both determine the biomass of global reef fish communities

Open Access This file is licensed under a Creative Commons Attribution 4.0 International License, which permits use, sharing, adaptation, distribution and reproduction in any medium or format, as long as you give appropriate credit to the original author(s) and the source, provide a link to the Creative Commons license, and indicate if changes were made. In the cases where the authors are anonymous, such as is the case for the reports of anonymous peer reviewers, author attribution should be to 'Anonymous Referee' followed by a clear attribution to the source work. The images or other third party material in this file are included in the article's Creative Commons license, unless indicated otherwise in a credit line to the material. If material is not included in the article's Creative Commons license and your intended use is not permitted by statutory regulation or exceeds the permitted use, you will need to obtain permission directly from the copyright holder. To view a copy of this license, visit http://creativecommons.org/licenses/by/4.0/.

Reviewer comments, first round review –

Reviewer #1 (Remarks to the Author):

This was an excellent body of work that takes worker heat exposure modelling to the next level. Quite complex methodology and data analysis. Conclusions have of course have limitations as would be expected by using surrogate measures to calculate WBGT. The most significant of this being the fact that globe temperature cannot be modelled and many workers are in fact exposed to direct sunlight. The paper does highlight this limitation.

One issue that might be considered in future work may be to use an alternative heat stress index, WBGT work-rest regime can be a bit over protective. In hot climates I do believe acclimatisation is a factor that leads to self pacing of work and many "hot" tasks are already scheduled for cooler parts of the day.

Congratulations on a very worthwhile body of work that draws attention to the cost of heat impacts.

Reviewer #2 (Remarks to the Author):

This paper builds on the latest models and projections of extreme heat under various climate change scenarios. It offers an essential analysis that goes beyond the extant literature which estimates of labour loss without adaptive measures, to offer an analysis of the effectiveness and limits of a particular adaption measure - that of rescheduling intensive or exposed work to cooler times of the day. This is an extremely valuable paper in that it enables basic estimates of the value (lost and gained) of this adaptation strategy in relation to hours of productivity and economic value. The paper builds on widely accepted metrics and methods, meaning that the findings are easily translatable and of immediate use to these policy arenas.

The paper has a few areas where it can be a little clearer or developed further to make its significance - which is substantial - more readily apparent, namely in a) how it understands extreme heat and heat stress and relates this to (s)WBGT and climatic conditions; b) the precise nature of the adaptation strategy it focuses on, and c) the implications of the limits it identifies in using this strategy - as part of loss and damage calculations and thereby as a platform for alternative adaptation options - and to support more informed and detailed decision-making about the portfolio and timing of adaptation strategies for countries, sectors and companies/organisations. d) where next - how this analysis can be used in more detailed scenario exercises and adaptation planning. [These points are detailed below.]

I have restricted the focus of my comments to the occupational health and productivity and climate adaptation framing and policy implications of this paper, as these relate to my fields of expertise. As non climate-scientist/lay reader of these aspects of the paper, I will note that it was one of the more accessible methods sections for such work that I have read, which I appreciated and think is valuable for the wider academic community.

Detailed comments:

a) The way the term 'humid heat' is used in the paper and its relationship to WBGT (and sWBGT) are a little unclear to me - I have the impression that (s)WBGT and humid heat are somewhat conflated/treated as synonyms and/or that WBGT is only of use for humid heat and not for dry heat.

For example, the commentary on Qatar suggests only humid heat presents a risk to workers, whereas it predominantly has hot and dry weather which also presents a risk. The value of WBGT (and similar) is that it enables us to interpret both dry and humid heat in a common way - i.e. in relation to the heat stress conditions (including low and high humidity) cause. This is the reason

why WBGT is useful for humid heat, as the heat stress this causes is typically under-represented by ambient Temperature, but it is also useful for more accurately assessing the heat stress caused by dry and hot conditions (which might otherwise be over-estimated).

As I understand it, because your paper assesses (s)WBGT, it is useful for understanding heat stress risk places that typically experience humid heat but also in places that experience dry heat, or both. The ability to compare risk in both dry and humid conditions is essential in adaptation planning either during the day or across season, for example in the mornings when temperatures are lower but WBGT could be higher - this is an additional reason why your analysis using sWBGT exposure and the benefit of work-time shifting is important, as assessments using ambient temperature only may have over-estimated the efficacy of this particular adaptation strategy.

Edits for clarity on the relationship between WBGT and humid heat would therefore present a more accurate picture of what your research does, and makes clearer its value for adaptation planning.

b) Time shift -vs- task shift. An edit for clarity and consistency throughout the paper on whether you mean shifting work hours (i.e. cessation of any kind of work/designation of non-working hours in the hottest day and moving those hours to a cooler period, changing the commencement and/or duration of the work day) OR shifting more exertional and/or exposed tasks from hotter hours to cooler hours, while retaining the original commencement and ending times of the shift would be helpful. Task-shifting/rescheduling might be seen as complementary to time-shifting, or there could be progression from one-to the other as climate change progresses. It would be great to spell out more clearly how your analysis supports weighing up these options.

c) Regarding the point above, although other heat management and adaptation strategies are mentioned, sometimes the paper seems to conflate time/task shifting with adaptation per se. Yes, overall adaptive capacity is limited by the limits to the efficacy of this particular option, but it would be good to see some more detailed examination of how that knowledge supports more robust adaptation planning - e.g.

-1: The limits of time/task shifting strategies and what it will cost in terms of lost productivity and GDP, calculated as stand-alone response. This could support arguments for funding alternative adaptation measures (e.g. through Warsaw International Mechanism for Loss and Damage).

- 2: choices between adaptation options, how to assemble a collection of strategies and weigh their utility including over time and under different scenarios (e.g. compensating for lack of value from time shifting by investing in active cooling interventions).

Minor comments -

Line 29 - the grammatical structure of the sentence implies avoiding unsafe working conditions *cause* labour productivity losses directly - whereas the latter usually refers only to a reduction in work rate, which may in fact be a sign of self-pacing and an appropriate response to heat. Suggest rephrasing (such as " ...unsafe and causes labour productivity losses ..'.

Line 42 - Consider costs as well as benefits, to avoid bias to positive outcomes.

Paragraph from Line 180 - heat strain impairing physical and cognitive function and contributing to higher accident and workplace injury rates is also worth mentioning - e.g. the just published study from Park, Pankratz and Behrer (2021).

269 - A consideration of formal (night) shift work would be valuable to consider in relation to your findings (e.g. swapping the 12 hottest hours for the 12 coolest hours). Large construction sites and resource extraction and processing are examples of heavy labour contexts where night shifts are often used (although usually in the context of 24-hour operations).

From a policy perspective, it is helpful to indicate in the main body/introduction to the paper that analysis of impacts is conservative, given use of ERA5 and basing the analysis on shade conditions

underestimates actual WBGT.

An indication of future research, and whether your analysis and data are available for use would be helpful.

Similarly, policies that restrict night shift/early morning work such as noise restrictions, industrial zoning etc should not be assumed to be permanent barriers to adaptation, but as triggers for a more extensive investigation of potential adaptation strategies and a reimagining of what a heat-adaptive society might look like.

The paper already makes a strong and useful argument, and provides valuable findings. With a bit more clarity on the above points it would offer an even more effective launch-pad for further research and more detailed adaptation planning by relevant policy communities.

Reviewer #3 (Remarks to the Author):

In this manuscript, Parsons et al. develop estimates of global labor productivity loss resulting from humid heat conditions in the present day as well in the future with different amounts of human-induced warming. The authors then quantify the percentage of productivity that could be recovered under several work-shifting scenarios that model changes in when heavy labor is performed within the standard 12-hour workday they modeled.

The questions the authors are asking with this analysis—namely, how will climate change affect the labor productivity of outdoor workers around the world? And how will the capacity to adapt to warming change as that warming grows more severe—are interesting ones. And they are important questions to be considering as nations head into the COP26 international climate negotiations, at which discussions of and commitments to reducing emissions and paying for the costs of climate adaptation take place. Thus, this paper is an important contribution to our collective understanding of the costs of climate change.

Overall, this is a strong piece of research. The manuscript is well written, the methods the authors employed were sound—though there are some comments below that I'd like to see addressed--and their conclusions followed reasonably from their results. Yet neither the methods nor the findings struck me as novel enough to warrant publication in Nature Climate Change given the caliber of the journal. For example, in performing their analysis, the authors essentially employed a previously published methodology for simulating futures with different amounts of warming relative the preindustrial era (i.e., those of Tigchelaar et al. 2020). And the core findings of increasingly severe heat constraints on labor are conceptually similar to the work of Dunne et al. 2013 and Kjellstrom et al. 2018, though it's notable that neither of those previous studies considered potential adaptation measures or adaptation capacity changes as the present manuscript does.

Comments:
1. In lines 55-57 and Figure 1, the authors describe locations where humid heat is already at or approaching levels unsafe for continuous heavy labor in the morning and at midday. In looking at the peak WBGT values in Figure 1, however, it's unclear what that unsafe level is and how it relates to the WBGTave presented in lines 245-246 of the methods section. From the methods section, I assumed that threshold would be 32.47C, but none of the locations in Figure 1 approach that value. I may be misunderstanding, but some clarity around what that threshold WBGT value is and how it relates to the equation in the methods section is needed. I suggest adding a sentence for clarity around lines 55-57.

2. The authors find that global labor losses due to extreme heat already more than 200 billion hours per year, with greater losses during anomalously or particularly hot years. Given that these findings are based on historical data, the manuscript would be strengthened significantly if they were vetted against an independent source of data. Are there any estimates—global or for any given nation—of actual labor productivity losses due to humid heat over the observational time period? As the authors note in the discussion, outdoor workers often choose to work during the

heat despite the health risks because they need the income. And I wonder if the losses calculated here for the historical period reflect what has actually transpired over that time period. Whether vetting the results with an additional data source is possible or not, I'd suggest future exploring in the text how well those historical results are capturing reality.

3. Lines 87-88: The authors find a strong relationship between temperature and labor loss, and while the relationship is very clean as presented in Figure 2, it is largely unsurprising given that the labor loss calculation is directly tied to WBGT as described in the methods. Perhaps some text in this section that more fully describes the importance of this finding would make the findings more compelling.

4. The analyses quantifying labor loss during the hottest and coolest hours of the day seem somewhat arbitrary. Is there evidence to suggest that workers are employers would deliberately shift heavy work in this manner rather than shifting a full workday to cooler hours? As it's currently presented, analyzing the potential benefit of shifting one hour of heavy labor to a cooler time of day seems more of a scientific exercise than a practical exploration of how work might actually shift in response to warming temperatures. The shifting of three hours, however, seems a much more likely occurrence. If there's evidence to suggest one-hour shifts in work time are taking place, that would be helpful to include in the section starting on line 166. If not however, I'd recommend trimming the paragraphs on the one-hour shifts and expanding the text on the three-hour shifts in work schedules.

5. Lines 257-261 (Methods): The authors assume that the 12-hour workday is evenly split, with four hours at the daily maximum WBGT, four hours at the daily mean WBGT, and four hours at the halfway point between the two. However, the data presented in Figure 1 seem to imply a different distribution of WBGT values over the course of a 12-hour workday. A reference is made to Kjellstrom et al. 2018, but it would be useful to include an explanation of whether or how the 12-hour WBGT data shown in Figure 1 supports this assumption.

6. Lines 328-331 (Methods): The explanation of why the 1%CO2 experiment was preferable to the more traditional emissions pathways from CMIP6 was somewhat unclear. The latter is said to have the potential to "create localized differences in the magnitude of warming," yet the authors then state that what they're interested in is local temperature changes. If the inclusion of non-CO2 greenhouse gases causes local temperature changes, would that not be important to include here? I'd like to see a more compelling or more clearly stated justification for using the 1%CO2 experiment.

7. Much of the methods section (particularly lines 364-398 or so) describes the temperature and humid heat warming patterns from CMP6 models. The results here are central to the study, as those warming patterns are applied to the reanalysis data in order to simulate future humid heat conditions, and while the choice to unpack them in the methods section is understandable, I'd suggest crafting a paragraph or two describing these findings at a high level and including that text in the main body of the paper.

8. Similar to my previous comment, the results described in the methods section seem overly detailed and like they are largely explaining what is presented in the supplementary figures. I'd suggest a) pulling some of this results-focused text from the present methods section into the supplementary information; and b) presenting a few concise metrics that describe these results in the methods section.

Reviewer #1 (Remarks to the Author):

Species richness and identity both determine the biomass of global reef fish communities

This research explores a large dataset of reef fish assemblages aiming to understand drivers of community biomass on reefs. The authors used an innovative approach, partitioning differences in fish biomass in five components. Establishing reference sites based on high biomass values, the authors performed thousands comparisons within a radius of 100 km from the reference sites. The authors found that richness loss is the most important driver of biomass, and that species' identities and their traits (body size) are key factors in predicting community status. Moreover the authors discuss that human population is the most important variable explaining species loss.

The paper in general is very interesting and well organized. It is also very relevant. There are many papers that show differences in biomass between sites and explore their drivers, but most very locally. This manuscript by Dr. Lefcheck and collaborators brings novel information in analyzing thousands of sites together and applying a new approach to better understand the effects of changes in biodiversity and environmental variables.

We thank this reviewer for their positive assessment of our work.

While I see the significance of the paper, and support its publication, I also see potential for improvements, mainly related to better clarifying the definitions of Richness and Compositional loss-gains. I am also concerned about the methods used to standardizing the number of transects in each site, once the paper deals with richness numbers. Please find below a few comments and suggestions to improve these and other points, which I believe most are not hard to address.

Sincerely,

Hudson T. Pinheiro

Line 34 – What is being driven? Biodiversity or Community biomass? Please clarify.

Clarified as "changes in community biomass."

Line 66 – Please clarify the relationship between RICH-L with the "expected" or average biomass per species. Are expected" and average biomass per species the same?

Clarified as "'based on the expected' (i.e., average)."

Line 67 – I believe that species absence is a best baseline because it is pure lost biomass by richness loss, am I right? So RICH-L is pure lost biomass by richness loss and lost biomass by difference with "average value", right? While COMPL-L (lines 69-71) is only the pure lost biomass by richness loss?

The RICH-L term considers the loss of biomass due to loss of species, but if all individuals of all species had the same average biomass (or, more precisely, that shared species had the same per species biomass on average as species that are absent from the focal site). So, it is the pure richness component independent of identity. The COMP-L term, on the other hand, summarizes the actual

loss in biomass AWAY from this average, therefore capturing contributions of very large or very small species that deviate from this average and are absent at the comparison site. We have clarified this text and hope this makes clear the proper interpretation.

Line 68 – Definition of average value is not very clear. Is the "average value" the average of biomass between the two compared communities? Or is it related to the reference community?

We have expanded this text to note that it is both reference and comparison communities (lines 70-74).

Lines 63-71 – You need to clarify the relationship between RICH-L and COMP-L, maybe rewording some sentences. Regarding a more technical point, did you thought about the beta-diversity components turn-over and nestedness when creating this novel partitioning method?

As noted above, we have made this relationship clearer in the text. We did not consider beta diversity in the traditional sense, since the equation partitions biomass and not diversity. We have not changed the text to note this to avoid confusion with the existing interpretations.

Line 73 – Although in my opinion RICH-G and COMP-G are much better defined (much easier to understand), I found incongruences between definitions of COMP-L and COMP-G. COMP-L says "… capture the unique contributions of species that are only found at the reference site" while COMP-G "…reflects the deviation of these gained species from the average contribution of shared species among the two sites". So, Is composition L or G related to species loss/gained or differences in biomass of shared species?

We agree that this explanation could be clearer. The "G" components merely consider the biomass associated with the gain of species at the reference site (or those species only found at the reference site). The compositional effect is determined again by the deviations from the expected gain based on the average biomass vs. the realized gain.

Line 76 – What is DIV? What is the justification to sum these values? What I am understanding so far is that COMP is part of the RICH component.

We have now defined DIV in the text as "the total effect of changing biodiversity between sites."

Line 78 – The definition of this term seems the same of COMP-G, I suggest merging with the next sentence that better explain differences.

Clarified that this does not consider loss or gain of species, as the other components do.

Line 90-92 – How did you select? What did you use for cut?

We ordered sites based on their total community biomass and conducted comparisons over the 100 km radius for each of these sites sequentially (omitting sites that were included in previous comparisons; see revised Methods and response to R2, below).

Line 102-105 – "the biodiversity effect was driven almost entirely by the loss" – But this was part of your method, when you chose to define sites with high biomass as reference. If you decided to analyze the gain, establishing poor sites as reference, the biodiversity would be driven by the gain…

We refer to the loss of species, not biomass. The equation does not require that the highest biomass sites also have the highest number of species: it just so happens that this is how these communities are structured in nature, and therefore why we witnessed a decline in biomass associated with a decline in species richness.

Line 108 – What do you mean with changes in species composition? Please clarify and standardize the term, because this remind me turn-over, but you only analyzed species loss/gain or differences in biomass in shared species. Same here in line 110 "changing species' identities"

We have clarified throughout that the "COMP" terms capture changes in community composition, and are therefore concerned with changes in the identities of the species between the two communities (rather than simply the total number of species). For example, communities can have identical richness but opposite compositions because the identities of the species are non-overlapping.

Line 266 – What is the richness expectation?

Clarified (lines 310-311).

Line 267 – "this term reflects the deviation in the actual contributions of lost species from the average of shared species" What term? Can you clarify this "deviation"

We refer the Reviewer to the discussion of δ_B and δ_F in the Methods (lines 287-289).

For me, the definitions are still confuse even in the methods. For example, line 258 shows "we interpret this term as the 'richness loss' or the loss in functioning due strictly to the loss of species: RICH-L", while in the line 269 "'compositional loss,' or the degree to which loss in biomass is due to loss of particular species: COMP-L. Both refer to loss of species, and what about biomass loss in shared species?

We have strived to clarify throughout the manuscript that the "RICH" terms consider only the number of species and average biomass of shared species, whereas the "COMP" terms additionally consider how the biomass of species unique to the reference or comparison community differs from the average biomass of shared species.

Line 318 – It is not clear how you determined the reference sites. What was he cut?

We have clarified this section (lines 364-369) to note that reference sites were determined by ordering the highest biomass sites and working sequentially through this list to conduct all comparisons within 100 km, while omitting any sites as reference sites that were included as comparison sites in previous comparisons.

Methods – general remarks

Please clarify the range of transect numbers for each location, and could you also add more environmental characteristics of the transects (info about habitats, depth range, etc). Moreover, how did you deal with difference in transect number between sites? Locations with more transects

should have more species and be responsible to differences.

We have clarified the methods to indicate that at any location where >1 transects were conducted, the biomass values were collapsed into a single average for each species. Nevertheless, the median number of surveys for a given location was 2, and the average number of surveys per comparison site was 3.2 compared to 2.9 for the reference site, suggesting that any upward bias in biomass and/or richness is equalized between the two. A slight replication bias would therefore be towards more species at the *comparison* sites, whereas we found more species, in general, at the reference sites, making our results potentially conservative.

Furthermore, the number of surveys per site was only weakly correlated with the components (e.g., RICH-L, COMP-L, etc.) for either the baseline site (mean correlation r = -0.3, range = [-0.19, 0.15]) and the comparison sites (r = 0.02 [-0.08, 0.13]), demonstrating that there is not a strong or systematic relationship between the degree of replication and the final elements of the decomposition.

We have also clarified the distribution of habitats (see response to Reviewer #2).

Reviewer #2 (Remarks to the Author):

This is an interesting paper that uses a large data set and sophisticated analysis to separate the effects of varying fish richness and identity on changes in biomass (a proxy for functioning). I agree with the authors that this is an important question, and rarely tested in marine ecosystems. Although the patterns (human populations drive down the biomass and richness of large-bodied species) are not novel, the paper details exactly what is changing within fish assemblages across anthropogenic gradients. My comments are relatively minor.

We thank this reviewer for their positive assessment of our work.

1) The authors are transparent that they are studying standing biomass, but they do switch between talking about biomass and ecosystem functioning (e.g. in the Abstract). I think the validity of using biomass as a proxy of functioning needs a bit more justification in the Introduction (e.g. the Duffy reference cited on line 49 refers to productivity rather than standing biomass). I don't doubt that biomass correlates with many aspects of functioning, but I think the text could make a stronger case. The Discussion might also mention that we need to eventually move to similar analyses with actual ecosystem functions as response variables.

This is a fair point, and we have made changes throughout the manuscript (Abstract, introduction) and added a statement in the discussion about extensions to true ecosystem functions (i.e., quantifying *rates* of key ecological processes).

2) I think the choice of sites needs clarifying, and would benefit from a map in the ESM. The paper talks about reference sites and surrounding comparison sites, but how were these designated. Was it a series of non-overlapping 100km circles from some random start point? Or slightly overlapping? Or was it 100km circles round the 173 sites with the highest biomass? This is important as in the latter case the sites could theoretically all be close to each other with large overlaps. What is the split between coral and rocky reefs?

Good point, and we have added the map of sites included in Figure S5.

We designated reference sites based on biomass (lines 364-369). We first ordered all sites in order of decreasing biomass. The top site (with very highest biomass) was then selected at the first reference site, all sites within 100 km of that site were used as comparisons in the decomposition, then these reference and comparison sites were removed from the ordered list. Then, the next highest biomass site (not involved in any previous comparisons) was selected, all sites within 100 km identified, and so on until all sites were considered as either reference or comparison sites. We also dropped any reference sites with <5 comparisons, as we felt that was too few to robustly apply the decomposition. We have updated the methods to better describe this procedure.

Out of the 3040 sites in total, 1437 sites were in temperate realms (predominantly rocky substrate) sites and 1603 sites were in tropical realms (mostly coral substrate), so the comparisons were evenly split between the two ecosystems.

3) It is good that the data set spans multiple ecosystems (coral and rocky reefs) to gain generic insights, but I think it would be worth looking at (maybe just in the ESM for completeness) whether the patterns are consistent between these ecosystems or whether the results represent an 'average' result. It is possible that different biophysical and social drivers in each ecosystem could lead to some interesting and useful differences.

This is an interesting idea and we were happy to explore it. When we parse the components of the decomposition by system, we find that the qualitative interpretations are the same, but there are some differences (see Figure S2).

For example, loss of biomass associated with loss of species (RICH-L) tended to be more extreme in tropical locations, while compositional losses showed the inverse, with more extreme contributions by large-bodied species in the temperate areas. These results track with current ecological theory: tropical sites tended to have almost 4x as many species as temperate ones (mean = 111 vs. 39.3), consistent with the latitudinal diversity gradient, and therefore allowing for a greater scope for species losses (RICH-L). By contrast, temperate sites tend to have larger individuals on average, with both a larger average per capita biomass (mean = 700 vs. 487) and an order of magnitude larger maximum per capita biomass. As a result, temperate sites stand to lose more extreme amounts of biomass with any given species, driving down COMP-L. Nevertheless, the total diversity component (DIV) is no different between the two, implying that these two trade-offs cancel each other out.

We have included this as a supplementary figure and make a brief mention in the text (lines 150-158).

4) I suggest listing all the random forest covariates in the Methods – at the moment you can only see the list of variables in Fig. S2. I was surprised not to see complexity as a variable – presumably the divers measured some estimate of habitat complexity during fish surveys? It is well established that fish respond dramatically to changes in complexity, so it seems a critical covariate to include in the random forests to fully explore biophysical influences on the results. Also, is there an explanation for why the nutrients might be so important in the models – is it just fueling primary productivity? Or maybe correlated with something else? There is also now a global market gravity

layer – this might be useful along with the population index data?

Good point, we now list all the covariates in the methods (lines 400-402).

The covariate selection for this study was determined by an exhaustive procedure previously detailed in Duffy et al. 2016 PNAS on the same dataset. This earlier study considered 25 variables ranging from the physical environment to human population to biological factors (such as complexity) and selected 12 to include in the analysis, which we use here (including temperature min and max). Complexity in the form of variables like coral and algal cover was not selected by this earlier screening due to low explanatory power and was further discounted due to a high degree of interpolation across the entire survey footprint as no consistent measure of structural complexity of the underlying reef structure is available for *all* surveys included in the dataset. We highlight that the CDE term theoretically accounts for these influences, and complexity may be a key underlying driver of the CDE term, albeit untestable for Fig S2. Importantly, we do not think this omission affects the conclusions of the study.

The Duffy study interpreted nutrients as an indicator of resource availability and primary production, although we note it was sufficiently uncorrelated with surface chl-*a* to be reasonably included in the linear models in that analysis. We would hold the same interpretation here, noting that in nearshore areas, it may also be an indicator of nutrient pollution and human activities that may or may not be correlated with human population. The global market gravity index is an interesting suggestion, although we have not yet worked with those data. Future analyses—specifically those focused on exploited species—could benefit from exploring this new metric.

5) It would be interesting to get a sense of whether the baseline sites are relatively 'natural' – i.e. are the comparisons to reefs close to their carrying capacity? For example, were any of the sites in well-established marine reserves where fishing mortality may be ~0, or are the values close to emerging metrics on unfished reefs of ~1000kg/ha (eg MacNeil et al, Nature 520:341-344).

This is an interesting question, but whether the sites are at carrying capacity is perhaps beyond the scope of the current study. It should not, however, have implications for the given study given that the method is inherently comparative.

The notion of marine reserves is an interesting one and a topic we explored early. Unfortunately, while the RLS dataset has explicitly targeted a number of MPAs, many of these are too remote to facilitate a large number of comparisons. As a result, the results were somewhat idiosyncratic, which is further compounded by the fact that these MPAs vary in many characteristics that are well-known to affect biomass (eg, size, enforcement, etc. see NEOLI by Edgar et al. 2014). As the dataset continues to grow to include more surveys in reserves, it is certainly worth revisiting this topic in a dedicated presentation.

Reviewer #3 and Reviewer #4 (Remarks to the Author):

Lefcheck Review

Overview
The authors develop a new partition of the ecological Price equation, which they use to analyze

biodiversity-ecosystem function relationships in coral reef fish communities. This work is impactful and will be of interest to many readers. We are excited to read the final version and think it will motivate new studies analyzing observational biodiversity-function data. The authors' most noteworthy result is that "changes in biodiversity" explain more variation in function among sites than "context dependence". We have a minor concern with how these verbal definitions map on to the math of the Price equation, but this is still a significant result because there are relatively few studies of the biodiversity-function relationship using worldwide, observational data. The work is original, with the caveat that their new Price equation partition was already considered (but not formally presented) in Fox 2006 Ecology. In our view, the authors have supported all conclusions and claims and provided enough detail for reproducibility.

We thank the reviewers for their positive assessment of our work.

Use of Price equation

We are not experts in coral reef communities, or even marine systems, so we have focused our comments on the authors' use of the Price equation and general issues in biodiversity-function research. We were impressed with and appreciated the thoroughness and curiosity the authors showed with respect to their data and Price equation partition. There are analyses over different scales, excluding different fish, a check on shared species numbers, a null model, etc. There was even a randomization test for the composition term. This paper is now among the most careful and thorough applications of the ecological Price equation that either of us have reviewed. There are just a few Price equation-related issues that need to be more clearly explained.

Again, we are glad that the reviewers appreciated our thorough approach to understanding and applying the Price equation.

Major comment

1. New Price equation partition:

This paper defines a five-part partition that is similar to but not the same as Fox & Kerr 2012. In both partitions, RICH-L + COMP-L sum to the total function of species lost from the baseline site. Both derivations are valid. Further, because the difference applies only to the RICH and COMP terms, the authors' main result about the importance of biodiversity vs. the context dependence effect is robust to this issue.

However, we are concerned because Fox 2006 explored and rejected a similar partition in Appendix 1 of Fox 2006 (Eq. A8) (the partitions are identical when no species are gained and per-species' function doesn't change). His conclusion was that the alternate partition was less desirable because, among other things, it confounded "the effect of loss of species richness per se with the effects of the processes that determine species' post-loss functional contributions". The new partition presented here uses a baseline community with pre-loss per-species functional contributions, but total baseline function is also affected by species presence/absence in the post-loss site, so the effect of species richness loss is confounded with a selection (ie composition) effect.

Neither of us are Jeremy Fox, nor do we in any way advocate treating Fox's Price equation work as

dogma. The Price equation is highly adaptable, and the authors' derivation may well represent an important new tool. But the justification for their choice of reference community is limited and the rationale is, at least to us, unconvincing (ll 309-313, see also line comments). We would really like to see the authors substantially expand on their justification for using a different reference community. Hopefully they can do this in a way that connects with the existing literature (i.e. Fox 2006 Appendix 1), although we don't consider this essential. Right now, this section is short, underdeveloped, and sticks out in a paper that is otherwise much stronger.

Thanks for these thoughtful comments. Indeed, there are numerous mathematically valid ways one could decompose the difference in biomass. In each decomposition, the resulting terms mean something different. Some of those decompositions result in more easily interpretable and/or biologically meaningful terms than others. Among the many options, we do not believe there is one "perfect" choice, though some are better than others for certain applications. We thought long and hard before making the choice we did. We agree our decomposition is imperfect but we believe it has some good properties. The one proposed by Fox (2006) is a good one too, but it is not the only good one and it has some issues as well. Fox (2006) only applies when the focal community has a strict subset of the species present in the reference community. That assumption was relaxed in the "extended Price equation decomposition" by Kerr and Godfrey-Smith (2009) that was employed in the context of community comparisons in the paper by Fox and Kerr (2012), hereafter FK12.

Like FK12, we are interested in decomposing total biomass into three kinds of components: (i) richness (i.e., biomass changes due to differences in the number of species between sites); (ii) composition (i.e., biomass changes due to differences in the types of species at two sites); and (iii) CDE (i.e., biomass changes due to differences in the biomass of the species that are present at both sites). To evaluate the "richness" component, it is necessary to have some basis for the "expected" change simply due to species loss (or gain) as if species where exchangeable (i.e., if species were "standard" species or came from some distribution of "standard" species). Essentially, the question becomes, how do you *choose* to define a "standard" species (i.e., what is the frame of reference)? We chose a different answer to that question than FK12.

We have little interest in criticizing the decomposition used by FK12 as that was an obvious inspiration for our work, and we actively avoided any such criticism in our original submission. However, in response to the reviewer's questions we need to point out that the FK12 decomposition comes with its own set of problems. Before going into a more full-blown theoretical comparison, it is worth providing two very simple examples that illustrate that the FK12 decomposition behaves oddly in some straightforward cases.

Example 1: The baseline and focal community have the same number of species (i.e., no difference in species richness) but differ in composition by species. Specifically, the baseline community has a unique species of high value (e.g., a population of a large apex predator) whereas the focal community has a unique species of only modest value (e.g., a population of a small predator)

**Species**	**Value in baseline community**	**Value in focal community**
Small_Sp1	20	20
Small_Sp2	25	25
Small_Sp3	30	30
Med_Sp1	50	50

Med_Sp2	absent	60
Large_Sp1	300	absent
TOTAL	425	185

Here are the decompositions side-by-side

**Term label**	**FK12**	**Ours**
RICH-L	-85	-31.25
RICH-G	+37	+31.25
COMP-L	-215	-268.75
COMP-G	23	+28.75
CDE	0	0
Total change (ΔT)	-240	-240

In this example, the number of species in the two communities is the same (i.e., no difference in species richness). In our decomposition, the net richness effect (= RICH-L + RICH-G) is zero. In the FK12, the net richness effect is -48. In our decomposition, the net composition effect (= COMP-L + COMP-G) is -240, which is equal to the total difference between the communities. In other words, our decomposition attributes all of the difference to a composition effect. This is arguably the correct interpretation of the difference here! In contrast, in the FK12 decomposition, the net effect of composition effects is -192; they attribute only 192/240 or 80% of the biomass difference to composition. The remaining 20% is attributed to a richness effect, which is a very undesirable outcome given that the two communities have, by design, exactly the same species richness.

<u>Example 2</u>: Same as Example 1, but with a few more species in common. Let's simply add the same three new species to each community and they have the same value in both places (values: 25, 25, 55).

Here are the decompositions side-by-side

**Term label**	**FK12**	**Ours**
RICH-L	-66.25	-32.86
RICH-G	+36.25	+32.86
COMP-L	-233.75	-267.14
COMP-G	23.75	+27.14
CDE	0	0
Total change (ΔT)	-240	-240

In our decomposition, the net richness effect remains 0 and the net composition effect remains -240. In the FK12 decomposition, the net richness effect has shrunk in magnitude to -30 and the net composition effect has grown in magnitude to -210 (but still only 87.5% of the total effect). In both examples, the true difference between the communities is entirely one of species composition and that difference in species composition is exactly the same in both examples. Yet, the magnitude of FK12's net composition effect differs between the examples.

Example 1 illustrated that FK12's decomposition resulted in terms *qualitatively* misrepresented the biology. Example 2 illustrates that the magnitude of the difference in biomass attributed to differences in species composition between communities also depends on the species that are shared, not just the species that are different. It would be misleading to pretend that our decomposition works in a perfectly sensible manner under all possible circumstances. While there can be strange outcomes in some cases, our decomposition performs sensibly under these basic scenarios, especially given the objectives of our article.

It is worth noting that the decompositions are simply different from one another so even in a simple case where both perform in a qualitatively sensible way, they give different quantitative values.

<u>Example 3</u>: This is the example given in the Appendix of Fox (2006). The baseline community has four species with values 1, 2, 3, and 4 respectively. The focal community is missing the first two species and the values of the last two species are 2 and 6.

Here are the decompositions side-by-side:

**Term label**	**FK12**	**Ours**
RICH-L	-5	-7
RICH-G	0	0
COMP-L	2	4
COMP-G	0	0
CDE	1	1
Total change (ΔT)	-2	-2

Qualitatively, the two decompositions are the same; both have a negative RICH-L term, positive COMP-L and CDE terms, and the other terms are zero. The terms differ quantitatively because the frame of reference for the decompositions are different.

We now walk through a more thorough mathematical comparison of the two decompositions. At the outset, it is worth recognizing that when comparing two communities there are three categories of species: (i) those unique to the baseline (or "reference") community; (ii) those unique to the focal (or "comparison") community; and (iii) those in common between the two communities. Everything about the total biomass of the two communities and difference between them (ΔT) depends on two simple properties for each category: the number of species in the category and the average biomass per species (represented in the table below). These simple properties are nice because they are so easy to understand. We have presented our decomposition in a way that emphasizes these simple properties.

Category Of species	Number in **baseline** site	Average biomass per species in **baseline** site	Number in **focal** site	Average biomass per species in **focal** site
Species *unique* to the **baseline** site	s_{uB}	$\bar{z}_{uB}$	NA	NA
Species *unique* to the **focal** site	NA	NA	s_{uF}	$\bar{z}_{uF}$
Species *common* to both sites	s_c	$\bar{z}_{cB}$	s_c	$\bar{z}_{cF}$

All species	$s_B = s_{uB} + s_c$	$\bar{z}_B = s_{uB}\bar{z}_{uB} + s_c\bar{z}_{cB}$	$s_F = s_{uF} + s_c$	$\bar{z}_F = s_{uF}\bar{z}_{uF} + s_c\bar{z}_{cF}$

Our decomposition results in 5 terms presented in the manuscript that are reasonably easy to interpret with respect to the simple properties in the table above. The presentation of the terms in FK12 are less so. The FK12 decomposition is presented (see their eq 1) as:

$$\Delta T = (s_c - s_B)\bar{z}_B + (s_c - s_F)\bar{z}_F + Sp(w^{!}, z) + [-Sp(w_j, z)] + \sum_{i=1}^{s_c} \Delta z_i$$

We have adjusted their notation slightly to make it more compatible with ours. Here $s_B = s_c + s_{uB}$ [total species number at the reference site (they use s)], $s_F = s_c + s_{uF}$ [total species number at the focal site (they use s')], $\bar{z}_B$ is the mean of *all* species at the reference site (they use $\bar{z}$), $\bar{z}_F$ is the mean of *all* species at the reference site (they use $\bar{z}'$), and $Sp(x,y)$ is the sum of products operator and they use w values as indicators of whether species is present at both sites (see their paper for details). The first two terms are what they refer to as their “richness” loss and gain terms. The next two terms are what they refer to as their “composition” loss and gain terms. The final term is their CDE. It is hard to get a feel for what these terms represent by looking at the equation the way they show it, especially those composition terms (terms 3 and 4). However, after doing some algebra one can express their 5 terms with respect to the simple properties that are easier to understand. Writing the terms in the same order as in the equation above gives

$$\Delta T = -s_{uB}\bar{z}_B + s_{uF}\bar{z}_F - \frac{s_c s_{uB}}{s_c + s_{uB}}(\bar{z}_{uB} - \bar{z}_{cB}) + \frac{s_c s_{uF}}{s_c + s_{uF}}(\bar{z}_{uF} - \bar{z}_{cF}) + s_c(\bar{z}_{cF} - \bar{z}_{cB})$$

What we call richness and composition terms differ from what they call those terms. The table below provides a side-by-side comparison of the two decompositions, using the same simple parameters:

**Term type**	FK12	Ours
RICH-L	$-s_{uB}\bar{z}_B$ $= -s_{uB}\left(\frac{s_c}{s_c + s_{uB}}\bar{z}_{cB} + \frac{s_{uB}}{s_c + s_{uB}}\bar{z}_{uB}\right)$	$-s_{uB}\bar{z}_{cB}$
RICH-G	$s_{uF}\bar{z}_F$ $= s_{uF}\left(\frac{s_c}{s_c + s_{uF}}\bar{z}_{cF} + \frac{s_{uF}}{s_c + s_{uF}}\bar{z}_{uF}\right)$	$s_{uF}\bar{z}_{cF}$
COMP-L	$-\frac{s_c}{s_c + s_{uB}}s_{uB}(\bar{z}_{uB} - \bar{z}_{cB})$	$-s_{uB}(\bar{z}_{uB} - \bar{z}_{cB})$
COMP-G	$\frac{s_c}{s_c + s_{uF}}s_{uF}(\bar{z}_{uF} - \bar{z}_{cF})$	$s_{uF}(\bar{z}_{uF} - \bar{z}_{cF})$
CDE	$s_c(\bar{z}_{cF} - \bar{z}_{cB})$	$s_c(\bar{z}_{cF} - \bar{z}_{cB})$ [same as FK12]

The CDE effect is the same in both decomposition and that means the sum of the remaining 4 terms (DIV = RICH-L + RICH-G + COMP-L + COMP-G) is also the same. However, the 4 individual terms differ between the decompositions. Our decomposition is premised on using the shared species as the frame of reference for "standard" species (discussed below). The expected loss of biomass due to number of species that are absent from the focal site (RICH-L) depends on the number of those species (s_{uB}) and average biomass of the "standard" species $\bar{z}_{cB}$. Our compositional effect directly captures the way those species absent from the focal community are different from standard (i.e., shared) ones ($\bar{z}_{uB} - \bar{z}_{cB}$), weighted by how many species are absent (s_{uB}) from the focal community. This seems a straightforward way to quantify the ideas of "richness" and "composition" effects.

Now compare the FK12 COMP-L effect to ours. It is the same as ours but multiplied by the fraction of species at the baseline site that are shared with the focal site, $s_c/(s_c + s_{uB})$. It eludes us why one would want to include this extra factor as it obscures biological interpretation. Rather, we suspect that extra factor was an unintended consequence of FK12 mathematical decomposition and they likely did not realize it because they did not express their terms with respect to the simple properties that we have.

Why are the two decompositions different? At one level, it is simply because they did the math differently than we did; both are valid, but different, mathematical routes that have the same starting place but lead to different end points. They were heavily inspired by the Price equation of evolutionary biology and the logic that underlay that route. While the idea of decomposing biomass differences was heavily inspired by their work, our approach to the decomposition was different. Our decomposition was driven by thinking about what we wanted the terms to represent biologically and making use of the simple properties that must underlie all the differences (the s and $\bar{z}$ values of the three categories of species).

From our vantage point, a key issue is the frame of reference. To evaluate the "richness" component, it is necessary to have some basis for the "expected" change due to a species loss (or gain). In other words, what is lost value from losing a single "standard" species? In our parlance, FK12 implicitly chose to represent a "standard" species (or, more accurately, the mean of the distribution of standard species) as the mean of **all** species at a site. In contrast, we chose to define a "standard" species by the mean of the **shared** species between sites. Both choices are arbitrary to some degree; neither is a perfect choice. The odd (and arguably undesirable) feature of the FK12 COMP-L term that we discussed above is really just a mathematical consequence of FK12 implicitly using all species as the basis for a standard species. This is what leads to their COMP-L term behaving in an undesirable fashion in the two simple examples we provided.

As discussed above, the choice of FK12 to use all species as the frame of reference complicates the interpretation of the compositional terms. Even if unique species contributed much more biomass per species than did shared species, the COMP term in the FK12 decomposition would go towards zero if there were many more unique species than shared species; the diversity loss is then *misleadingly* attributed to a richness effect. For the current study, we want the COMP terms to capture the consequences of unique species being different from shared species (with respect to average biomass). In the JK12 decomposition, that type of effect is muddied by the relative numbers of unique and shared species due to the averaging over all species as their reference set.

To address these problems, our decomposition uses the shared species as the reference set, so we then evaluate the average value of unique species with respect to its deviation from the average

value of shared species. This approach explicitly separates the contributions of shared and unique species. One of the nice features of our decomposition is that the ratio of COMP to RICH is independent of the number species, which seems sensible given both are quantifying effects from the same species (number and type) absent from the focal site; it directly captures the compositional effect (scaled to the expectation), i.e., whether or not unique species are more valuable in terms of biomass contribution per species than are shared species, which is our central question. (Our decomposition: COMP-L/RICH-L $= (\bar{z}_{uB}/\bar{z}_{cB}) - 1$; FK12 decomposition: COMP-L/RICH-L $= s_c(\bar{z}_{uB} - \bar{z}_{cB})/(s_c\bar{z}_{cB} - s_{uB}\bar{z}_{uB})$.)

Lastly, we want to address this particular statement from the reviewers who wrote that Fox

"…rejected a similar partition in Appendix 1 of Fox 2006 (Eq. A8) (the partitions are identical when no species are gained and per-species' function doesn't change). His conclusion was that the alternate partition was less desirable because, among other things, it confounded "the effect of loss of species richness per se with the effects of the processes that determine species' post-loss functional contributions". "

First, Fox's (A8) is not the full decomposition so it is hard to say how similar it is; he only breaks it into two terms and then decides to abandon that approach rather than continuing to split the second term. Even if we focus on the first term, as the reviewers note, it only becomes the same as ours under particular conditions. The reviewer's quote of Fox's concern is about this first term. It isn't clear to us what the phrase quoted from Fox (2006) by the reviewers means precisely. We believe it refers to the situation when per-species' function changes between sites (the subsequent text in Fox suggests so). As the reviewer notes, the first term of Fox's (A8) is the same as ours when one assumes that such changes do not happen. The two decompositions (or even the first terms of each) are not the same when species' function do change between sites. In other words, Fox's concern applies to his (A8) in a case where his (A8) and our decomposition are different.

One additional biological justification for our choice to use shared species as the frame of reference is that we *know* shared species can exist in either community because *they do exist* in both communities! Species that are unique to one location may not be capable of persisting at other location for a variety of reasons and, thus, may be "special" (i.e., not standard) in some sense. We realize counter arguments could be made. We would not claim our "biological justification" constitutes an unassailable rationale for doing it this way, but we would argue that is a *reasonable* way to do it. We think the resulting terms in our decomposition are easier to understand and typically do a better job of quantifying what people intuitively mean when they think of "richness" or "composition" effects.

Much of the preceding content (i.e., the mathematical comparison of our decomposition and FK12) is now presented in Appendix A.

Line comments

ll 59-60 check refs 11-15: the expectation for baseline per-species function is calculated from just the shared species, rather than the whole baseline community. This decision and its justification needs to be briefly but clearly conveyed early in the main body of the paper as well as the detailed explanation in the methods.

See above: we have also updated the manuscript to include a condensed version of the above explanation as an appendix and now referenced on lines 74 and 359 so readers can understand the

justification behind the choices for this particular decomposition.

ll 79-82 This phrasing is maybe misleading. The environment and population dynamics (and the sampling scheme) presumably drive species turnover between sites, and also (potentially) affects the abundance and body sizes of all species at a site, whether or not they occur at other sites. So environment etc. potentially drives ALL five terms (as you know, and very nicely illustrate in Fig S2, and discuss concretely in ll 127-145). I get a similar feeling about ll 99-102, which is really a core claim for the paper. I think just being a bit more precise about the wording would be helpful, together with a clearer explanation of your conceptual model for the relationships between environmental drivers, biodiversity, and CDE.

In this same section, you define the CDE as "all factors other than biodiversity". Is this really a good way to define this term? Change in abundance and relative abundance seem to fall well within common definitions of "biodiversity". If a community lost or gained no species, but shifted from highly even to dominated by one species, is this not a change in "biodiversity"? It's a minor issue, but there may be a clearer way to capture what RICH+COMP means for readers who are not inclined to dig into the math. We don't have a better suggestion, unfortunately, so we are fine with the authors keeping the current term if they don't think of an improvement.

We agree with the reviewers that the interpretation of the CDE term is complicated and these individual drivers cannot be cleanly separated from other components. We have updated the language in these paragraphs to better clarify what CDE captures and how it can be interpreted with respect to our results, referring back to the original language of Fox (2006) and Fox and Kerr (2012).

l 85 not sure what "direct" means here

Removed.

l 144 why "indirectly" responsible?

The most likely pathways for these effects are by changing resources and/or environmental conditions (the fishes are not responding directly to phosphorus but by stimulation of primary production, for example), but removed for clarity.

ll 185-186 No, the Price equation when applied to observation data does not say anything about complementarity. The Loreau & Hector citation only applies to experiments that control abundance (ie area) and have monoculture data for each species. The diversity effects captured by the Price equation are strictly additive. Any non-additive effects of diversity, like complementarity, are hopelessly mixed with environment/any other number of effects.

We agree, thanks, and clarified.

ll 262-263 Please clarify that for each pairwise comparison, you are dividing all five terms by the absolute value of the largest magnitude term.

Done (also repeated later in the Methods).

ll 263-264 This is good information and thanks for including it. But it is more convincing is a "our results are not affected" sense than in a "this won't be an issue for this partition in the future" sense.

Agreed, and we have noted this.

ll 311-313 Why does the "average" species need to be common? We know communities have few common and many rare species, so unless function is decoupled from abundance, the mean (or median) function across species is likely closer to the rare species than the common species.

Agreed, we have removed this language.

l 363 "implicit bias" inadvertently references social psychology jargon. I think a one-phrase reminder of the bias you are testing for would be more informative here.

Replaced with "a consequence of our new decomposition."

l 365 you state in the methods (l 170) that you hold per-capita function constant, but here "per species biomass" implies per-capita x abundance (ie species totals)

The per capita biomass was retained from the raw data, but the per species could change depending on how the new abundances are randomly re-assigned.

l 369 do you include 0s in these averages? If you do, large species that occur infrequently will appear small (ie presence-absence is conflated with per-capita function)

This is an excellent point and upon revisiting the simulation code, we found that we were indeed erroneously including zeroes in calculating the averages. We have removed 0's from the calculations and re-run all simulations. The qualitative inferences remain unchanged.

ll 372-387. I am very happy you are using a randomization/simulation to understand the effect of a certain composition structure/chance effect on your Price partition values! But, your randomization/simulation is complicated and I'm not sure how it works. It would be great if you could state clearly (~2 sentences) what your simulation holds fixed, what it breaks, and why that gives you insight into whether your observed composition effect is large (the null is that sites with higher richness are more likely to include unique high-mass species, right?). Also, you need to explain why a two-tailed test is relevant here; some of your observed values are closer to 0 than the null distribution (RICH-L, RICH-G and COMP-G; see comments for l 580)

We have now edited the methods to make the goal of these simulations clearer as well as the how they were performed. The main goal of these simulations is to test if the observed negative COMP-L effect was simply an artifact of assigning the higher biomass site as the "reference" community and the other lower biomass site as the "focal" community. We show that the observed COMP-L effect is much larger in magnitude than any bias that would creep in from this assignment if communities were assembled at random.

We agree that including a formal statistical test here is valid, so we have implemented a one-tailed test (to account for the statement that the observed values are more extreme, ie more negative) using the means and standard deviations from the observed and simulated data and now report them in the text.

The simulation you describe randomizes presence-absences, not with column shuffles (preserving richness and community structure, but breaking association between composition and species' traits ie characteristic biomass), but by randomizing the presence-absence vector within each site. This breaks both the association between composition and the species biomass distribution, but also all other compositional structures other than richness differences (ie patterns of nestedness and modularity will be lost). Then, abundances are chosen at random from a uniform distribution, capped at each species' max value. I'm not sure what this will do to the abundance distribution.

We agree with the reviewer's characterization of the simulations. However, we do not have any reason to believe this will affect our primary goal here. As we have now clarified, the primary goal of these simulations is to try to test whether the compositional effect we observe is an artifact of how we assign which site is the reference community and which is the focal community. We are assembling communities at random with respect to composition while using observed values for species richness and realistic values for their abundances. This seems a reasonable approach to the issue of concern (i.e., whether a large negative COMP-L term is simply an artefact of our choice of reference community).

l 500 maybe a typo, do you mean z_uB - z_cB? and then for the composition gain term, z_uF - z_cF. The term z_uC is ambiguous as to whether it refers to the mean function of shared species in site F or site B.

Fixed.

l 511 should read "resulting in a non-zero CDE". Also the following sentence "In this example…" has some kind of typo; perhaps you could just delete it, since the next starting with "In a real comparison…" is clear on its own.

Done.

l 567 "vastly increase" does not seem to match the modest x-axis ranges of these plots. Do you consider any of these variables to be highly influential? Anyways, I really like Fig S2, and you cite it several (3?) times throughout the paper; this makes me wonder if it deserves to be included as a main-text figure.

Removed "vastly." We considered including Fig. S2 in the main text, but we believe the salient points are captured in Fig. 3.

l 580 It is true that they all fall outside their null distribution, but the observed values have smaller magnitude than the null distribution (RICH-L, RICH-G, COMP-G) as well as larger (COMP-L, CDE, overall DIV). I'm not sure what this means, but "more extreme" isn't accurate.

Looking at figure S4 - you are right that their observed values fall outside the null value distributions. At the same time, (1) I'm struck by how much of the overall story can be recaptured by assuming random community assembly and (2) the observed values have smaller magnitude than the null distribution (RICH-L, RICH-G, COMP-G) as well as larger (COMP-L, CDE, overall DIV). What does this mean?

We have changed this to reflect that the observed means are “significantly different” than the simulated values based on the one-tailed test described above.

The overall story is recovered because we are simply redistributing existing per capita contributions to generate new compositions/biomass among the existing sites. Greater deviations from these patterns would be expected, for example, if we allowed species richness to vary as well, but this becomes complicated quickly and does not address our primary question, as noted above.

Reviewer comments, further review -

REVIEWERS' COMMENTS

Reviewer #1 (Remarks to the Author):

Dear Dr. Lefcheck and colleagues,

Thank you for the good job reviewing the paper. It sounds much clearer now. I have few more questions this time:

Line 29 -31 - "... the degree to which this relationship depends on the identities rather than the number of the species being lost remains untested at broad scales" - This is not true because we know well about the functional over-redundancy in reef fishes, where the addition of new species hardly adds new functions - see papers from Mouillot group, especially the 2014 PNAS paper.

Line 152-154 – In my opinion, having a higher richness in tropical reefs is not a reason for a more extreme decline in biomass per richness. It is because the higher richness in the tropics is mainly driven by small species (Barneche et al 2019 among others). Therefore, I would expect the opposite pattern, with a stronger decline in temperate systems where larger species predominate.

Sincerely yours
Hudson Pinheiro

Reviewer #2 (Remarks to the Author):

The authors have satisfactorily answered all my concerns, with the minor exception of them referring to a map in the Supplementary Materials that I could not see after downloading. Otherwise, I have no further concerns about the manuscript.

Reviewer #3 (Remarks to the Author):

Major comments

My main concern with the initial submission was the authors' choice to mathematically redefine the richness and composition terms. The authors have done an outstanding job responding to this concern, providing a six-page-long response including tables and demonstration analyses. Their response was thoughtful and informative, and revealed some drawbacks of the "standard" five-term ecological Price equation (Fox and Kerr 2012 or FK12) that I had not considered.

The authors' changes to the main text, e.g. at L70-76, are also very good.

As the authors themselves point out, neither the authors' approach, nor FK12, is perfect for biodiversity research. But both are reasonable choices, and the work should still be published. Further, the authors' main result is robust to this entire issue, as it compares RICH+COMP to CDE.

Minor comments (many of these don't necessarily require any changes)

Throughout: Reading this paper again, I feel like the role of abundance is maybe under-discussed? Not changes in the abundance of shared species as captured by the CDE, but just aggregate abundance. Since it's not the focus of your paper, I'm not sure there's much to do about this, other than maybe noting that patterns like baseline sites having more richness might arise simply because baseline sites have more abundance.

L29, L46, and throughout: I wonder if "biodiversity loss" is the best framing, given what your method accomplishes. Your approach also captures the effects of gains, and there is a long-running debate about how much biodiversity is declining vs. simply changing in nature (Vellend, Dornelas, Gonzalez, etc.). I have no issue with keeping the "biodiversity loss" framing but I worry that it undersells the types of community change that you can see in observational data and analyze with your method.

L41-43: a bit of an odd sentence since the idea of losing large-bodied species applies to very few BEF studies

L51-54: or the possibility that higher richness is simply associated with higher abundance in observational data, although I concede that's digressive for the paper as a whole

L54-58: I just want to say, this is such as great system for asking the questions that you have chosen

L70-76: Excellent, concise justification for your new partition. L346-358 are also good on this point.

L81-89, and L319-327: Perhaps more could be said about gains here. Are species gains controlled by an identifiable, interesting process? Or, not to be too informal, are they not very important and just there to make the math add up?

L141: Yes, if anything the effect of COMP is *enhancing* RICH in this case

L171: Is there a missing word here? Maybe "does not exclude species entirely"? Otherwise I'm confused

L215-221: Very nice and useful section

Reviewer #1 (Remarks to the Author):

Dear Dr. Lefcheck and colleagues,

Thank you for the good job reviewing the paper. It sounds much clearer now. I have few more questions this time:

We thank the reviewer for their positive assessment of our revision.

Line 29 -31 - "... the degree to which this relationship depends on the identities rather than the number of the species being lost remains untested at broad scales" - This is not true because we know well about the functional over-redundancy in reef fishes, where the addition of new species hardly adds new functions - see papers from Mouillot group, especially the 2014 PNAS paper.

This is a good point, we have revised to note "taxonomic identities" to avoid confusion with functional identity (line 29).

Line 152-154 – In my opinion, having a higher richness in tropical reefs is not a reason for a more extreme decline in biomass per richness. It is because the higher richness in the tropics is mainly driven by small species (Barneche et al 2019 among others). Therefore, I would expect the opposite pattern, with a stronger decline in temperate systems where larger species predominate.

We agree that larger species in temperature systems will contribute more to biomass declines (which is stated on lines 153-156). As for tropical coral reefs, we are referring specifically to biomass loss associated with the richness loss component, which is a function of the average or expected species (irrespective of whether the fishes are small or large). Therefore, we expect this component to capture more of the biomass change at tropical sites with more species.

Sincerely yours
Hudson Pinheiro

Reviewer #2 (Remarks to the Author):

The authors have satisfactorily answered all my concerns, with the minor exception of them referring to a map in the Supplementary Materials that I could not see after downloading. Otherwise, I have no further concerns about the manuscript.

We thank the reviewer for their positive assessment of our revision. Apologies for the oversight on the map, that has now been included in the Supplementary Materials (Fig. S6).

Reviewer #3 (Remarks to the Author):

Major comments

My main concern with the initial submission was the authors' choice to mathematically redefine the richness and composition terms. The authors have done an outstanding job responding to this concern, providing a six-page-long response including tables and demonstration analyses. Their response was thoughtful and informative, and revealed some drawbacks of the "standard" five-term ecological Price equation (Fox and Kerr 2012 or FK12) that I had not considered.

The authors' changes to the main text, e.g. at L70-76, are also very good.

As the authors themselves point out, neither the authors' approach, nor FK12, is perfect for biodiversity research. But both are reasonable choices, and the work should still be published. Further, the authors' main result is robust to this entire issue, as it compares RICH+COMP to CDE.

We really appreciate these positive comments.

Minor comments (many of these don't necessarily require any changes)

Throughout: Reading this paper again, I feel like the role of abundance is maybe under-discussed? Not changes in the abundance of shared species as captured by the CDE, but just aggregate abundance. Since it's not the focus of your paper, I'm not sure there's much to do about this, other than maybe noting that patterns like baseline sites having more richness might arise simply because baseline sites have more abundance.

L29, L46, and throughout: I wonder if "biodiversity loss" is the best framing, given what your method accomplishes. Your approach also captures the effects of gains, and there is a long-running debate about how much biodiversity is declining vs. simply changing in nature (Vellend, Dornelas, Gonzalez, etc.). I have no issue with keeping the "biodiversity loss" framing but I worry that it undersells the types of community change that you can see in observational data and analyze with your method.

Good point, we have changed this to read: "Biodiversity change alters ecosystem functioning" in the abstract, and discuss both "losses and gains" later in the intro (line 48).

L41-43: a bit of an odd sentence since the idea of losing large-bodied species applies to very few BEF studies

True, but this is arguably a question of logistics rather than importance: large-bodied species most assuredly contribute greatly to the ecosystems in question (see review by Estes et al. 2018 on Marine megafauna).

L51-54: or the possibility that higher richness is simply associated with higher abundance in observational data, although I concede that's digressive for the paper as a whole

Indeed, although application of rarefaction (as in the Duffy et al. 2017 PNAS study) could alleviate some of the issues with abundance and/or sampling effort.

L54-58: I just want to say, this is such as great system for asking the questions that you have chosen

We like to think so too!

L70-76: Excellent, concise justification for your new partition. L346-358 are also good on this point.

Thank you!

L81-89, and L319-327: Perhaps more could be said about gains here. Are species gains controlled by an identifiable, interesting process? Or, not to be too informal, are they not very important and just there to make the math add up?

In this application, it's the losses that are strongest and of most interest, so it does become a bit of mathematical convenience. In other applications, perhaps less so, especially as papers (eg, Vellend et al and Dornelas et al) demonstrate local gains in terrestrial systems.

L141: Yes, if anything the effect of COMP is *enhancing* RICH in this case

Not clear, perhaps you mean the loss of biomass formerly associated with COMP is now in RICH? Not necessarily, the distribution of RICH values actually gets closer to zero (but not by a lot) with decreasing scale.

L171: Is there a missing word here? Maybe "does not exclude species entirely"? Otherwise I'm confused

Good catch, fixed.

L215-221: Very nice and useful section

Thank you again!
